# The AUTACE That Degrades KRAS and Engages CD8^+^ T Cells for the Treatment of KRAS/TP53 Co‐Mutant Tumors

**DOI:** 10.1002/advs.202518455

**Published:** 2026-04-07

**Authors:** Luo Li, Bolin Li, Yanan Hao, Bo Pang, Pan Li, Chunli Li, Yu Tang

**Affiliations:** ^1^ Department of Laboratory Medicine Women and Children's Hospital of Chongqing Medical University Chongqing China; ^2^ Department of Laboratory Medicine Chongqing Health Center for Women and Children Chongqing China; ^3^ Department of Immunology College of Basic Medicine Chongqing Medical University Chongqing China; ^4^ Department of Ultrasound the First Affiliated Hospital of Chongqing Medical University Chongqing China

**Keywords:** autophagy‐targeting degraders, biomimetic nanovesicles, kras/tp53 co‐mutations, t cell engagers, tumor immunotherapy

## Abstract

KRAS and TP53 co‐mutations are frequently associated with highly aggressive, therapy‐resistant cancers with limited treatment options. In this study, we have developed Autophagy‐Targeting Chimera–T‐cell Engager (AUTACE), a bifunctional nanoplatform composed of T‐cell receptor–engineered T (TCR‐T) cell–derived nanovesicles that display anti‐CD3 antibodies and encapsulate perfluoropentane (PFP) together with KPY, an autophagy‐targeting degrader active against mutant KRAS, for the treatment of KRAS/TP53 co‐mutant tumors. AUTACE targets tumors via TP53‐specific TCRs, elicits antitumor CD8^+^ T‐cell responses through surface anti‐CD3 antibodies, and employs low‐intensity focused ultrasound (LIFU) to trigger controlled release of KPY to degrade mutant KRAS. This achieved targeted tumor elimination. The therapeutic efficacy of AUTACE was validated in mice bearing PANC‐1 and MIA PaCa‐2 tumors. A comprehensive assessment of the post‐treatment tumor microenvironment revealed that KRAS degradation increased tumor‐derived CCL5 levels, thereby promoting CD8^+^ T‐cell recruitment and amplifying antitumor responses. Thus, AUTACE represents a promising strategy for the treatment of KRAS/TP53 co‐mutant tumors.

## Graphical abstract

AUTACE is a bifunctional nanoplatform that integrates tumor targeting, immune engagement, and on‐demand KRAS degradation. It targets KRAS/TP53 co‐mutant tumors via TP53‐specific TCRs, elicits antitumor CD8^+^ T‐cell responses through surface anti‐CD3 antibodies, and uses low‐intensity focused ultrasound (LIFU) to trigger controlled release of the KRAS degrader KPY. By concurrently suppressing KRAS signaling and activating adaptive antitumor immunity, AUTACE effectively controls KRAS/TP53 co‐mutant tumors.

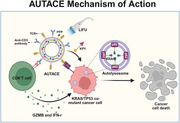

## 1 Introduction

Co‐mutation of KRAS and TP53 defines a subset of highly aggressive tumors such as pancreatic ductal adenocarcinoma (PDAC) [1, 2], colorectal cancer (CRC) [3, 4], and non‐small cell lung cancer (NSCLC) [5, 6]. Although KRAS/TP53 co‐mutant NSCLC exhibits improved responsiveness to immune checkpoint inhibitors (ICIs) [7, 8, 9], co‐mutant PDAC [10, 11, 12] and CRC [13, 14] generally derive little benefit from ICIs. Although the KRAS^G12C^ inhibitor sotorasib has demonstrated meaningful clinical efficacy [15], its application is limited to a small subset of patients because of its lack of activity against the more prevalent KRAS^G12D^ and KRAS^G12V^ oncogenic variants [16]. Additionally, clinical development of direct TP53‐targeting therapies remains at an early stage, and their therapeutic potential has yet to be established [17, 18]. Furthermore, mutant KRAS and TP53 cooperate to drive tumor progression [19], making single‐pathway inhibition insufficient to arrest tumor growth. These observations underscore the urgent need to explore alternative therapeutic strategies for KRAS/TP53 co‐mutant tumors.

Targeted protein degradation (TPD) utilizes endogenous cellular machinery in order to degrade disease‐causing proteins, thereby achieving more sustained pathway suppression than traditional inhibition [20, 21, 22]. Recent developments in pan‐KRAS degraders offer a potential strategy to target a range of oncogenic KRAS variants [23, 24, 25]. However, translational progress with these degraders is limited by the challenge of ensuring sufficient delivery to tumor tissues [26], highlighting the need for effective tissue targeting and controlled release.

T‐cell receptor‐based T‐cell engagers (TCR‐TCE) are bispecific constructs consisting of a soluble TCR that recognizes tumor peptide–HLA complexes and an anti‐CD3 antibody fragment that recruits and activates CD8^+^ T cells [27, 28, 29]. Consequently, engineering TCR‐TCEs in order to deliver TPD payloads is a promising approach for achieving spatially controlled delivery while simultaneously eliciting T‐cell‐mediated antitumor immunity. However, conventional antibody–drug conjugation (ADC) strategies are suboptimal for such constructs, as they typically allow only low drug‐to‐antibody ratios and bulky payloads can compromise TCR‐TCE activity [30, 31, 32], thus highlighting the need for refined designs that preserve TCR‐TCE function while supporting higher TPD loading.

Based on these considerations, we constructed Autophagy‐Targeting Chimera–T‐cell Engager (AUTACE), a nanoparticle platform that integrates TCR‐TCE and TPD functionalities, in which TP53‐specific TCRs and anti‐CD3 antibodies are displayed on the nanoparticle surface, whereas perfluoropentane (PFP) and KPY, a mutant KRAS–targeting degrader, are encapsulated within the core. In KRAS/TP53 co‐mutant tumor models, AUTACE exhibited excellent tumor‐targeting performance and potent direct tumor cell killing by engaging CD8^+^ T cells and degrading KRAS through LIFU‐triggered KPY release. Concomitantly, KRAS downregulation increases tumor‐derived CCL5 levels, thereby recruiting additional CD8^+^ T cells and amplifying their antitumor responses.

## 2 Results

### 2.1 Engineering KPY: A Degrader Targeting Mutant KRAS

**FIGURE 1:**
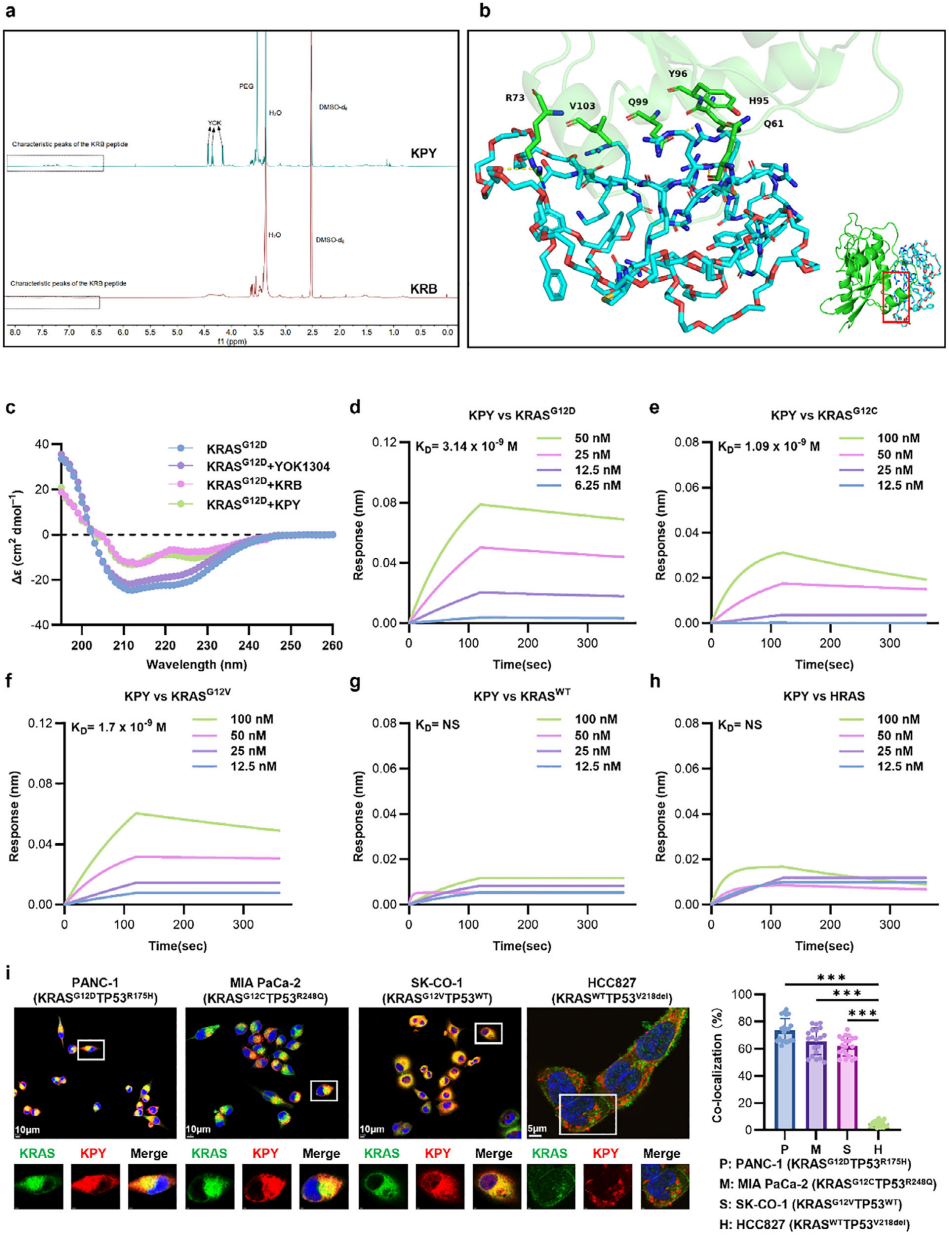
KPY binds mutant KRAS proteins. (a) Proton nuclear magnetic resonance was used to confirm the chemical structure of KRB and KPY. (b) Molecular docking model of KPY with KRAS G12D. (c) Circular dichroism spectra of KRAS G12D mixed with different formulations. (d–h) Biolayer interferometry was used to measure the affinity of KPY for multiple proteins; representative data from one of three independent experiments are shown. (i) Representative confocal microscopy images and quantitative analysis of the co‐localization between PE‐labeled KPY and intracellular KRAS in different cell lines after incubation with PE‐labelled KPY (20 µm) for 12 h (*n* = 20 cells per group). Data are presented as mean ± s.d.; statistical significance was assessed by one‐way ANOVA (i); ^***^ *p* < 0.001.

We first modified the structure of the KRpep‐2d (KR) peptide, which specifically binds to the mutant KRAS proteins [33, 34], by introducing lysine residues (K) at both the N‐ and C‐termini. A biotin moiety (B) was then conjugated to the C‐terminal K, yielding a modified peptide designated KRB (Figure 1a; Figure S1). Subsequently, we synthesized KPY, a degrader‐targeting KRAS mutant. KPY was constructed by linking KRB to YOK‐1304, which engages in p62‐dependent selective autophagy by binding to the p62 ZZ domain [21], using a polyethylene glycol 2000 (PEG_2000_) linker (Figure S2a–c). Mass spectrometry revealed a predominant peak for KPY at the theoretical molecular weight (*m/z* = 5800) (Figure S2d). When compared with KRB, the proton nuclear magnetic resonance (Proton NMR) spectrum of KPY retained the characteristic peaks of KRB and displayed additional signals corresponding to PEG and YOK‐1304, thus confirming that KRB was successfully conjugated to YOK‐1304 via a PEG_2000_ linker to form KPY (Figure 1a).

Several approaches were used to evaluate the interaction between KPY and KRAS mutants (G12D, G12C, and G12V) and wild‐type (WT) KRAS. First, molecular docking simulations predicted that KPY (colored ball‐and‐stick) forms a stable interface with key residues (Q61, H95, Y96, Q99, R73, and V103) of KRAS^G12D^ (green) through hydrogen bonding and hydrophobic interactions (Figure 1b), which is consistent with published reports [35]. Second, circular dichroism (CD) spectroscopy showed that upon the addition of KPY, mutant KRAS proteins displayed a pronounced upward shift between 208 and 222 nm, whereas WT KRAS remained unchanged (Figure 1c; Figure S3a–c), indicating the preferential binding of KPY to mutant KRAS. We also found that both KPY and KRB, but not YOK‐1304, significantly altered the secondary structure of KRAS^G12D^ (Figure 1c), indicating that the KRB moiety mediates the specific recognition of mutant KRAS proteins. Third, biolayer interferometry (BLI) assays revealed that KPY bound KRAS^G12D^, KRAS^G12V^, and KRAS^G12C^ with comparable affinities in the 10^^−9^ M range, while showing no detectable binding to WT KRAS or HRAS (Figure 1d–h). Across mutant KRAS, the affinities of KPY were found to be similar to that of KRB, demonstrating that conjugation to YOK‐1304 did not appreciably affect the binding affinity conferred by KRB (Figure 1d–h; Figure S3d–h). Finally, we examined the interaction of KPY with mutant KRAS in the cells. KPY showed dose‐dependent uptake in all tested cell lines by flow cytometry (Figure S3i–k), and immunofluorescence revealed strong co‐localization with the endogenous mutant KRAS in PANC‐1, MIA PaCa‐2, and SK‐CO‐1 cells, but not in the KRAS‐WT line HCC827 (Figure 1i). Collectively, these results have demonstrated that KPY specifically and effectively binds to multiple KRAS mutants (G12D, G12C, and G12V), providing a mechanistic foundation for subsequent autophagy‐mediated KRAS degradation.

### 2.2 KPY Induces Autophagy‐Dependent Degradation of Mutant KRAS

**FIGURE 2:**
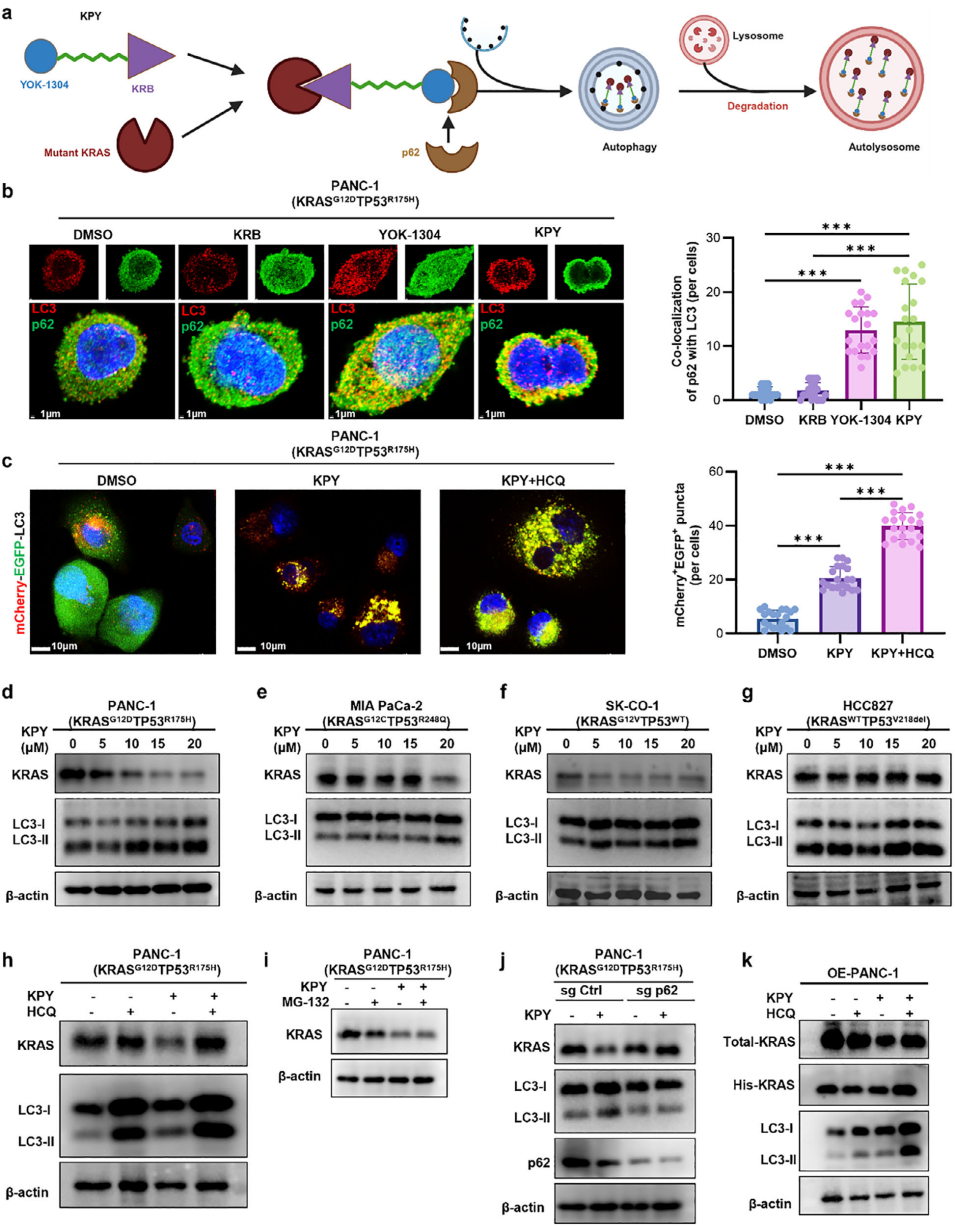
KPY induces autophagy‐dependent degradation of mutant KRAS. (a) Schematic of KPY‐mediated degradation of mutant KRAS. (b) Representative confocal microscopy images and corresponding quantitative analysis of LC3 and p62 co‐localization in PANC‐1 cells after treatment with KRB, KPY, or YOK‐1304 (20 µm each) for 12 h (*n* = 20 cells per group). (c) Representative confocal images and corresponding quantitative analysis of EGFP‐LC3 and mCherry‐LC3 co‐localization in PANC‐1 cells stably expressing mCherry–EGFP–LC3 after treatment with KPY (20 µm) alone or in combination with HCQ (10 µm) for 12 h (*n* = 20 cells per group). (d–g) Western blot analysis of KRAS, LC3, and β‐actin in different cell lines treated with increasing concentrations of KPY for 24 h. (h) Western blot analysis of KRAS, LC3, and β‐actin in PANC‐1 cells treated with KPY (20 µm), HCQ (10 µm), or the combination for 24 h. (i) Western blot analysis of KRAS and β‐actin in PANC‐1 cells treated with KPY (20 µm), MG‐132 (1 µm), or the combination for 24 h. (j) Western blot analysis of KRAS, LC3, p62, and β‐actin in PANC‐1 cells with sgRNA‐mediated knockdown of p62 treated with KPY (20 µm) for 24 h. (k) Western blot analysis of KRAS, His‐KRAS, and β‐actin in PANC‐1 cells overexpressing His‐tagged WT KRAS (OE‐PANC‐1) treated with KPY (20 µm), HCQ (10 µm), or the combination for 24 h. For all western blot data, representative images from one of three independent experiments are shown. Data are presented as mean ± s.d.; statistical significance was assessed by one‐way ANOVA (b, c); ^***^ *p* < 0.001.

According to our design, the mobilization of p62‐dependent selective autophagy is required for mutant KRAS degradation (Figure 2a). Immunofluorescence analysis revealed that both KPY and YOK‐1304, but not KRB, induced the formation of LC3 puncta and increased p62‐LC3 co‐localization (Figure 2b; Figure S4a). Notably, no significant differences were observed between KPY and YOK‐1304 in these autophagy‐related quantitative measures, thus indicating that KPY promotes p62‐dependent selective autophagy primarily through its YOK‐1304 moiety (Figure 2b; Figure S4a). Using the tandem mCherry–EGFP–LC3 reporter, we quantified autophagic flux. Relative to the control, KPY increased mCherry^+^ EGFP^+^ (yellow) LC3 puncta; upon co‐treatment with hydroxychloroquine (HCQ)—a lysosomal function inhibitor that blocks late‐stage autophagic flux—yellow puncta further accumulated, indicating that KPY enhances autophagic flux (Figure 2c). We also observed that KPY promoted the co‐localization of KRAS with both LC3 puncta, p62, and the lysosomal marker LAMP1 in KRAS‐mutant cell lines (Figure S4b–d). These data suggest that KPY mobilizes p62‐dependent selective autophagy and promotes the sequestration of mutant KRAS into autophagosomes followed by lysosomal degradation.

Next, we assessed the ability of KPY to degrade the KRAS protein. KPY reduced mutant KRAS protein levels in PANC‐1, MIA PaCa‐2, and SK‐CO‐1 cells (Figure S5a). Although both KPY and YOK‐1304 increased LC3 II accumulation, reflecting elevated autophagy, YOK‐1304 did not decrease mutant KRAS levels (Figure S5a). In contrast, KRB neither enhanced LC3 II conversion nor reduced mutant KRAS levels (Figure S5a). These results indicate that the ability to degrade mutant KRAS requires the simultaneous presence of KRB and YOK‐1304 in the same chimera.

To further characterize the degradation behavior of KPY, we examined the concentration and time dependence of KRAS clearance. In the PANC‐1 cells, KPY promoted mutant KRAS degradation in a concentration‐dependent manner, with significant effects at 10 µm and near‐maximal effects at 15 µm (Figure 2d). Similar concentration‐dependent reductions in mutant KRAS were observed in MIA PaCa‐2 and SK‐CO‐1 cells (Figure 2e,f). Notably, despite robust LC3 II induction, KPY failed to reduce KRAS protein levels in the KRAS‐WT HCC827 line (Figure 2g). In addition, KPY induced the time‐dependent degradation of mutant KRAS, with a pronounced reduction observed at 12 h, which persisted for at least 24 h (Figure S5b).

Mechanistically, the inhibition of lysosomal function with HCQ, but not proteasomal inhibition with MG132, effectively blocked the KPY‐induced degradation of mutant KRAS (Figure 2h,i; Figure S5c,d). Moreover, the CRISPR/Cas9‐mediated knockdown of p62 abolished KPY‐induced LC3 II accumulation and mutant KRAS degradation (Figure 2j; Figure S5e). In order to further assess the selectivity of KPY for mutant KRAS, we overexpressed His‐tagged WT‐KRAS in the PANC‐1 cells (OE‐PANC‐1) (Figure S5f) and probed total KRAS and His‐KRAS separately. KPY reduced total KRAS while leaving His‐KRAS unchanged, and co‐treatment with HCQ restored total KRAS, indicating the specific depletion of mutant KRAS over WT‐KRAS by KPY (Figure 2k). Collectively, these data establish that KPY selectively degrades multiple KRAS mutants via p62‐dependent autophagy while sparing WT KRAS.

### 2.3 Antitumor Effects and Mechanistic Profiling of KPY In Vitro

**FIGURE 3:**
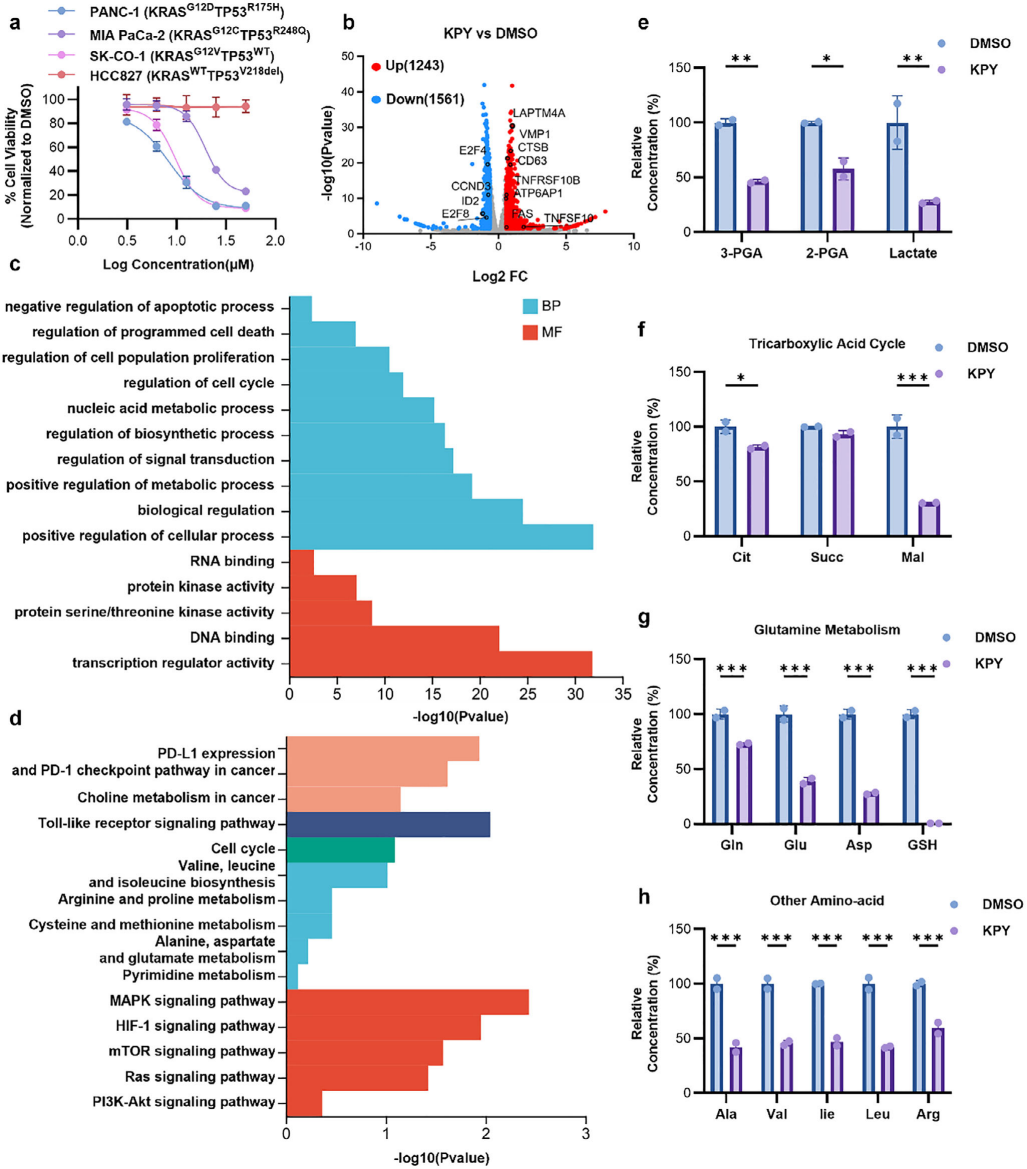
Antitumor effects and mechanistic profiling of KPY in vitro. (a) Cell viability of different cell lines treated with the indicated concentrations of KPY for 48 h, measured by the CCK‐8 assay (*n* = 3 per group). (b–d) RNA‐seq analysis of PANC‐1 cells treated with DMSO or KPY (20 µm) for 24 h: volcano plot of differentially expressed genes (b), GO (c), and KEGG (d) enrichment analyses of downregulated genes. e‐f) Metabolic changes of PANC‐1 cells treated with DMSO or KPY (20 µM) for 24 h: glycolysis (e), tricarboxylic acid cycle (f), glutamine metabolism (g), and other amino‐acid metabolism (h) (*n* = 2 per group). Data are presented as mean ± s.d.; statistical significance was assessed by two‐way ANOVA (e–h); ^*^ *p* < 0.05, ^**^ *p* < 0.01, ^***^ *p* < 0.001.

As KRAS mutations constitutively activate MAPK and PI3K signaling to promote tumor proliferation and survival [36, 37], we anticipated that degrading mutant KRAS would exert stronger cytotoxic effects on KRAS‐mutant cells than on KRAS‐WT cells. Indeed, KPY significantly inhibited the growth of PANC‐1, MIA PaCa‐2, and SK‐CO‐1 cells with different KRAS mutations, while exhibiting minimal effects on the KRAS‐WT line HCC827 (Figure 3a). Consistently, KPY selectively increased apoptosis in KRAS‐mutant cells, but not in KRAS‐WT cells (Figure S6a). In order to determine whether mutant KRAS degradation is causally linked to KPY‐induced cytotoxicity, we examined the effects of KPY on p62‐deficient cells. Consistent with our earlier observation that p62 depletion abolished KPY‐induced mutant KRAS degradation (Figure 2j; Figure S5e), CRISPR/Cas9‐mediated knockdown of p62 also abrogated the inhibitory effects of KPY on cell proliferation and abolished KPY‐induced apoptosis (Figure S6b–e). These results indicate that KPY‐mediated selective cytotoxicity depends on the clearance of mutant KRAS.

To profile transcriptional changes following KRAS degradation, we performed RNA sequencing (RNA‐seq) of PANC‐1 cells treated with DMSO or KPY. Relative to DMSO treatment, KPY treatment resulted in 1,243 upregulated and 1,561 downregulated genes. Volcano plots and Gene Ontology (GO) enrichment analyses indicated the upregulation of pro‐apoptotic genes and the downregulation of proliferation and cell‐cycle programs (Figures 3b,c). Kyoto Encyclopedia of Genes and Genomes (KEGG) pathway analysis further revealed the coordinated downregulation of oncogenic pathways, including the Ras–MAPK, PI3K–Akt/mTOR, and HIF‐1 pathways, as well as immune/checkpoint pathways (PD‐L1/PD‐1; Toll‐like receptor), cell‐cycle programs, and cancer metabolism (Figure 3d). Given the central role of KRAS in metabolic reprogramming [38, 39, 40], we assessed the effect of KPY on the metabolome of PANC‐1 cells. KPY reduced glycolytic and tricarboxylic acid (TCA) cycle intermediates, suggesting suppression of energy metabolism (Figure 3e,f). Likewise, metabolites in the glutamine axis, particularly reduced glutathione (GSH), were diminished, indicating impaired cellular redox buffering (Figure 3g). In addition, amino acid pools linked to proliferation decreased, indicating reduced biosynthetic capacity (Figure 3h). Taken together, these findings provide preliminary indications that the targeted degradation of mutant KRAS by KPY inhibits the growth and survival of KRAS‐mutant cancer cells by downregulating oncogenic signaling and broadly suppressing cancer metabolism.

### 2.4 Antitumor Efficacy of KPY In Vivo

**FIGURE 4:**
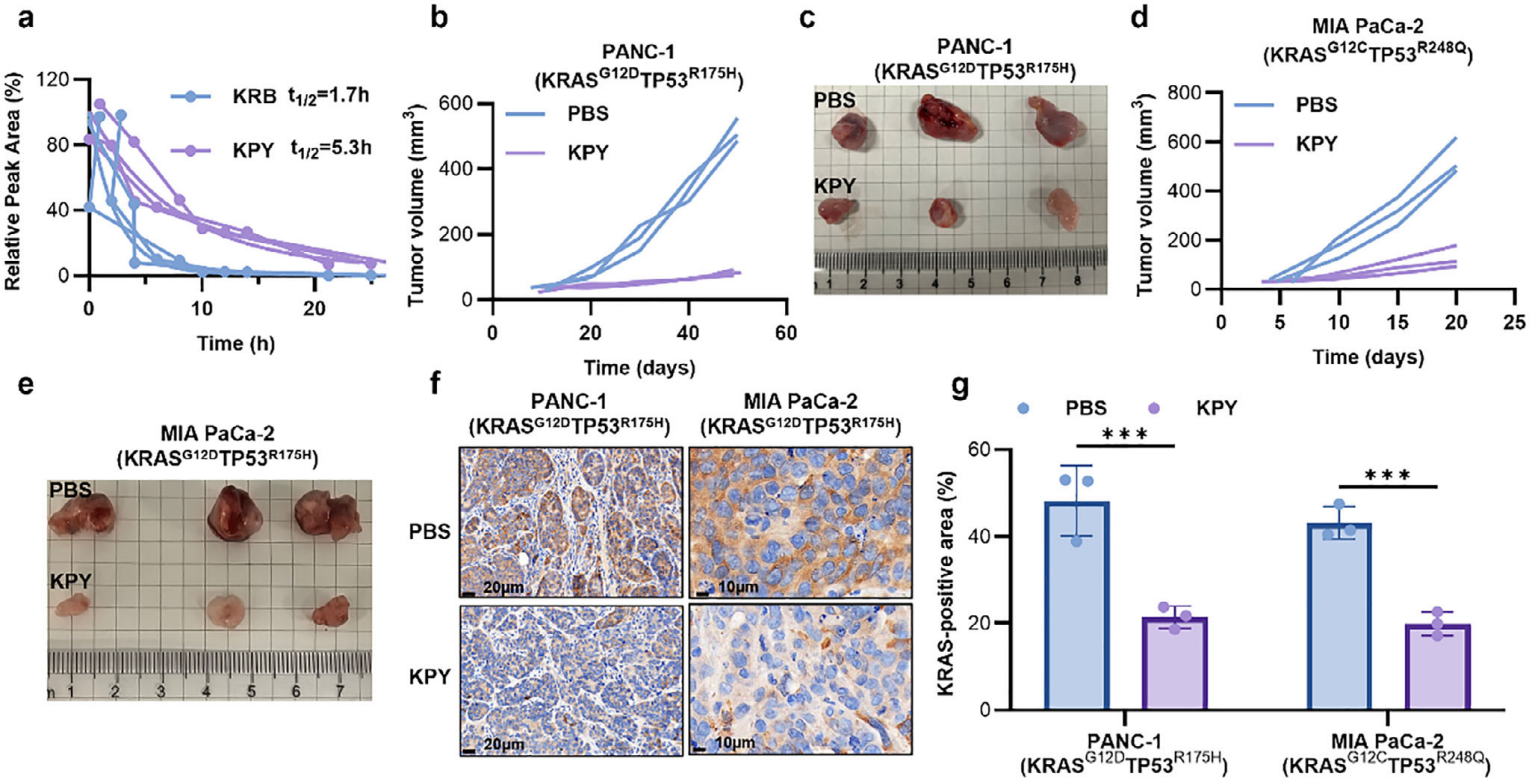
Antitumor efficacy of KPY in vivo. (a) Plasma stability of KPY and KRB determined by ultra‐high‐performance liquid chromatography–mass spectrometry (*n* = 3 per group). (b,c) Tumor growth curves (b) and tumor images (c) of PANC‐1 xenografts treated by intratumoral PBS or KPY (5 mg/kg) (*n* = 3 per group). (d,e) Tumor growth curves (d) and tumor images (e) of MIA PaCa‐2 xenografts treated by intratumoral PBS or KPY (5 mg/kg) (*n* = 3 per group). (f,g) Representative IHC images (f) of KRAS expression and corresponding quantitative analysis (g) in PANC‐1 tumors collected at day 50 and MIA PaCa‐2 tumors collected at day 20 after tumor inoculation in the indicated treatment groups (*n* = 3 per group). Data are presented as mean ± s.d.; statistical significance was assessed by two‐way ANOVA (g); ^***^ *p* < 0.001.

To assess whether the KPY‐mediated inhibition of tumor cell growth observed in vitro translated into potent antitumor efficacy in vivo, we evaluated its antitumor effects in subcutaneous xenograft models. Due to the instability of peptides in plasma, we first assessed the plasma stability of KPY and its precursor, KRB, by ultra‐high‐performance liquid chromatography–mass spectrometry (UHPLC–MS). Compared with KRB, KPY exhibited a markedly prolonged plasma half‐life, indicating that PEG_2000_ and YOK‐1304 conjugation substantially improved plasma stability (Figure 4a). Despite this improvement, intravenous (i.v.) administration of KPY did not significantly inhibit PANC‐1 tumor growth (Figure S7a). Immunofluorescence analysis revealed no detectable KPY within the tumors after i.v. dosing (Figure S7b), suggesting rapid degradation or clearance in the circulation, resulting in insufficient tumor exposure. Consequently, we adopted an intratumoral (i.t.) administration route to ensure adequate local exposure. Following i.t. injection, KPY was effectively retained in the tumor tissue (Figure S7b) and significantly inhibited the growth of both PANC‐1 (Figure 4b,c) and MIA PaCa‐2 xenografts (Figure 4d,e). This antitumor efficacy correlated with a marked reduction in KRAS protein levels in the tumors (Figures 4f,g). In contrast, KPY did not affect the growth of the KRAS‐WT HCC827 xenografts (Figure S7c). In summary, these data demonstrate that KPY exerts antitumor activity specifically in KRAS‐mutant tumors in vivo and that adequate local exposure is essential for effective pharmacodynamic engagement.

### 2.5 Preparation and Characterization of AUTACE

To overcome the limited efficacy of systemically administered KPY and achieve tumor‐targeted delivery, we incorporated KPY into a TCR‐T cell‐derived nanovesicle platform, AUTACE. We formed an AUTACE shell by fusing T cell membranes with liposomes. As a proof of concept, we labeled liposomes with Cy5.5 and membranes with FITC. Absorbance spectra, together with a detectable fluorescence resonance energy transfer (FRET) signal and confocal colocalization, verified successful membrane–liposome fusion (Figure S8a–c). Flow cytometry further showed that more than 90% of nanoparticles were FITC^+^Cy5.5^+^ double positive (Figure S8d). These concordant readouts indicated efficient membrane–liposome fusion.

**FIGURE 5:**
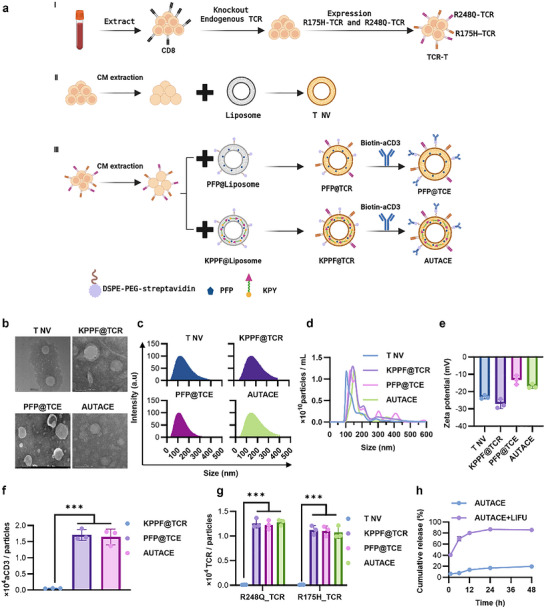
Preparation and characterization of AUTACE. a) Schematic of AUTACE construction. (b) Representative transmission electron micrographs of T NV, KPPF@TCR, PFP@TCE, and AUTACE. (c,d) Size distributions determined by dynamic light scattering (c) and nanoparticle tracking analysis (d). e) Zeta potentials of the indicated nanoparticles (*n* = 3 per group). (f) The number of anti‐CD3 per KPPF@TCR, PFP@TCE, and AUTACE, quantified by flow cytometry (*n* = 3 per group). (g) The number of R248Q‐specific and R175H‐specific TCRs per T NV, KPPF@TCR, PFP@TCE, and AUTACE, quantified by flow cytometry (*n* = 3 per group). (h) KPY release from AUTACE with or without LIFU treatment (*n* = 3 per group). T NV: liposomes fused with plasma membranes from T cells lacking TCR expression. KPPF@TCR: KPY‐ and PFP‐loaded liposomes fused with TCR‐T cell plasma membranes. PFP@TCE: PFP‐loaded liposomes fused with TCR‐T cell plasma membranes and conjugated with anti‐CD3 antibodies. AUTACE: KPPF@TCR further conjugated with anti‐CD3 antibodies. Data are presented as mean ± s.d.; statistical significance was assessed by one‐way ANOVA (f) and two‐way ANOVA (g); ^***^ *p* < 0.001.

The AUTACE fabrication process is illustrated in Figure 5a. Briefly, human CD8^+^ T cells were edited to eliminate endogenous TCR expression and subsequently transduced with TCRs specific for TP53 R175H [41] and R248Q [42] to generate TCR‐T cells (Figure 5a‐I; Figure S9). T NV was constructed by fusing liposomes with plasma membranes derived from T cells lacking endogenous TCR expression (Figure 5a‐II). Plasma membranes isolated from TCR‐T cells were fused with streptavidin‐liposomes encapsulating perfluoropentane (PFP) alone or co‐encapsulating KPY and PFP to generate PFP@TCR or KPPF@TCR, respectively (Figure 5a‐III). Finally, biotinylated anti‐CD3 antibodies were conjugated to the surface of PFP@TCR or KPPF@TCR via streptavidin–biotin interactions to produce PFP@TCE or AUTACE, respectively (Figure 5a‐III). Transmission electron microscopy (TEM) revealed the vesicular morphology of all nanoparticles (Figure 5b). T NV, PFP@TCE, KPPF@TCR, and AUTACE exhibited comparable hydrodynamic diameters with a stepwise increase following sequential drug loading and antibody conjugation, consistent with the progressive addition of functional components (Figure 5c; Table S1). Importantly, all nanoparticles maintained relatively narrow size distributions, with polydispersity index (PDI) values ranging from 0.12 to 0.26, indicating preserved colloidal stability during stepwise assembly (Table S1). Nanoparticle tracking analysis showed that all nanoparticles had particle concentrations exceeding 1 × 10^10 particles per milliliter (Figure 5d). Zeta potential analysis demonstrated that conjugation of anti‐CD3 antibodies increased the surface charge of PFP@TCE and AUTACE (Figure 5e). Quantitative analysis showed no significant difference in the mean copy number of anti‐CD3 per particle between PFP@TCE and AUTACE (Figure 5f). Similarly, the mean copy numbers of TCR were comparable among KPPF@TCR, PFP@TCE, and AUTACE (Figure 5g). The coefficients of variation (CVs) of CD3 and TCR copy numbers across the corresponding nanoplatforms ranged from 50% to 60% (Table S1), indicating appreciable particle‐to‐particle variability in CD3 and TCR surface density. The drug loading and encapsulation efficiencies of KPY in AUTACE were 4.38% and 68.75%, respectively. Basal KPY release from AUTACE was minimal, whereas low‐intensity focused ultrasound (LIFU) irradiation triggered a rapid, burst‐like release, enabling spatiotemporal control of payload delivery (Figure 5h).

### 2.6 Immune‐Activating and KRAS‐Degrading Effects of AUTACE In Vitro

**FIGURE 6:**
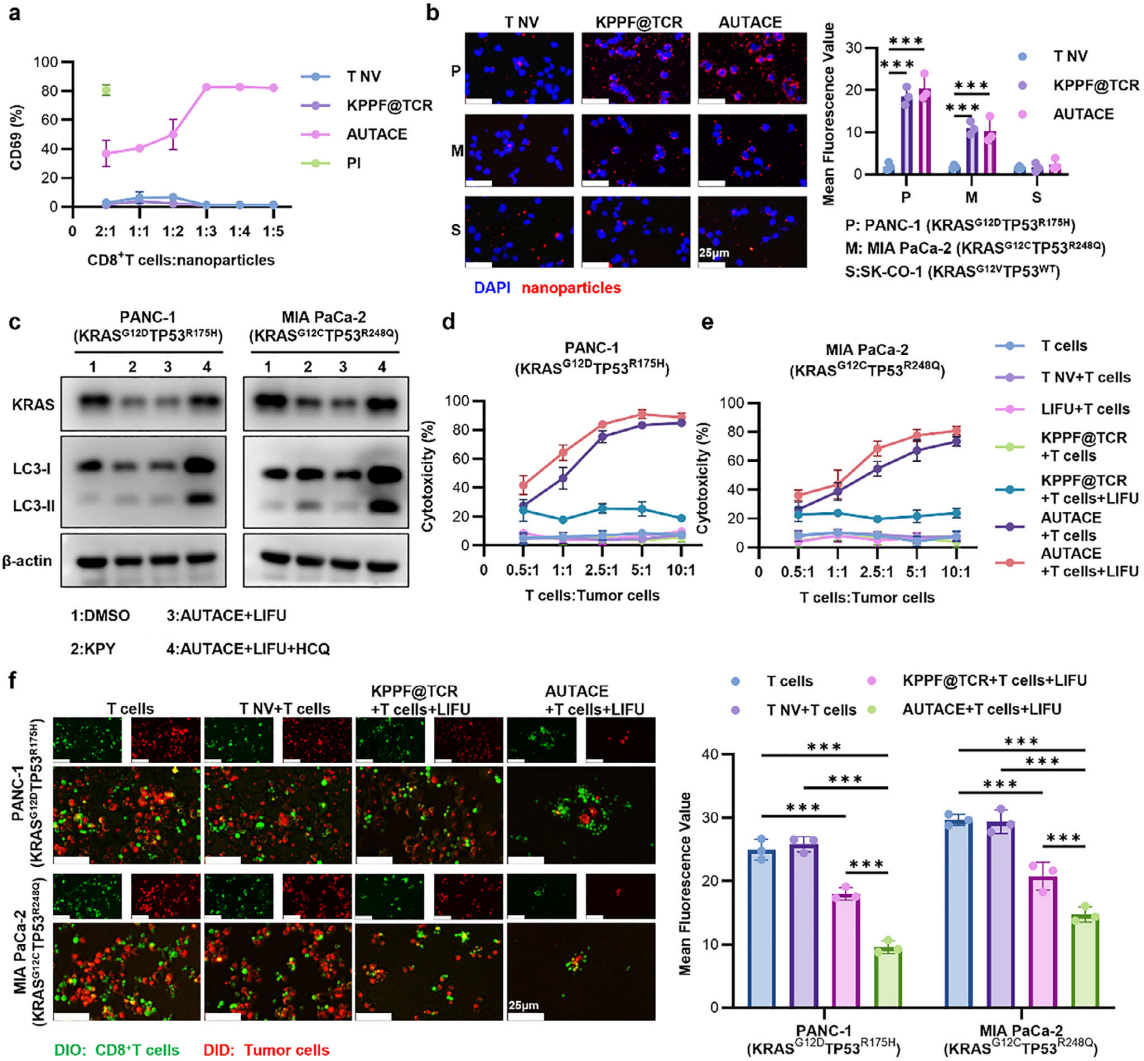
Immune‐activating and KRAS‐degrading effects of AUTACE in vitro. (a) CD8^+^ T cells were incubated with the indicated nanoparticles for 24 h, and CD69 expression was quantified by flow cytometry (n = 3 per group). (b) Representative confocal microscopy images and corresponding quantitative analysis of the binding of the indicated nanoparticles to tumor cells (*n* = 3 per group). (c) Western blot analysis of KRAS, LC3, and β‐actin in PANC‐1 and MIA PaCa‐2 cells treated with KPY (20 µm), AUTACE (3 mg/mL) plus LIFU (1 W/cm^2^ for 5 min), or AUTACE (3 mg/mL) plus LIFU (1 W/cm^2^ for 5 min) and HCQ (10 µm) for 24 h; representative images from one of three independent experiments are shown. (d,e) The survival of PANC‐1 (d) and MIA PaCa‐2 (e) cells was assessed by luciferase activity after 24 h of co‐incubation with CD8^+^ T cells and the indicated nanoparticle‐based treatments (3 mg/mL), with or without LIFU (1 W/cm^2^ for 5 min) as indicated (*n* = 3 per group). (f) Representative confocal microscopy images of tumor cells and corresponding quantitative analysis of fluorescence intensity after 24 h of co‐incubation with CD8^+^ T cells and the indicated nanoparticle‐based treatments (3 mg/mL), with or without LIFU (1 W/cm^2^ for 5 min) as indicated (*n* = 3 per group). PI: PMA+ Ionomycin. T NV: liposomes fused with plasma membranes from T cells lacking TCR expression. KPPF@TCR: KPY‐ and PFP‐loaded liposomes fused with TCR‐T cell plasma membranes. AUTACE: KPPF@TCR further conjugated with anti‐CD3 antibodies. Data are presented as mean ± s.d.; statistical significance was assessed by two‐way ANOVA (b,f); ^***^ *p* < 0.001.

Based on the above characterization, we next investigated the in vitro functionality of AUTACE. To determine whether AUTACE can activate CD8^+^ T cells in an anti‐CD3 antibody–dependent manner, human CD8^+^ T cells were incubated with the indicated nanoparticles. Compared with CD3 antibody–free controls (T NV and KPPF@TCR), AUTACE induced the robust activation of CD8^+^ T cells, as evidenced by the upregulation of CD69 and increased production of interferon gamma (IFN‐γ) and granzyme B (GZMB) (Figure 6a; Figure S10a–c). These results indicate that AUTACE activates CD8^+^ T cells through anti‐CD3 antibodies. To determine whether AUTACE recognizes tumor cells via TCR‐dependent interactions, various nanoparticles were incubated with different tumor cell lines. Immunofluorescence revealed that, relative to the negative‐control T NV, both KPPF@TCR and AUTACE exhibited increased binding to PANC‐1 and MIA PaCa‐2 cells harboring TP53 R175H or R248Q mutations, respectively, while exhibiting minimal binding to the TP53‐WT line SK‐CO‐1 (Figure 6b). The performed flow cytometry showed similar trends to those of immunofluorescence (Figure S10d). In addition, AUTACE failed to bind to PANC‐1 cells lacking the TP53 R175H mutant and to MIA PaCa‐2 cells lacking the TP53 R248Q mutant (Figure S10e). These data indicate that AUTACE specifically recognizes TP53 R175H and R248Q mutant tumor cells via TCR‐mediated interactions. We then assessed the degradation activity of AUTACE in mutant KRAS. Upon LIFU irradiation, AUTACE reduced KRAS protein levels in PANC‐1 and MIA PaCa‐2 cells to an extent comparable to that of free KPY at matched doses, and this effect was abolished by co‐treatment with HCQ (Figure 6c). In contrast, AUTACE without LIFU had little effect on KRAS levels (Figure S10f). Furthermore, mutant KRAS degradation was observed only in KPY‐loaded nanoparticles (KPPF@TCR and AUTACE) following LIFU irradiation, whereas KPY‐free controls (T NV and PFP@TCE) showed no detectable reduction in KRAS levels, even under LIFU exposure (Figure S10f). These results demonstrate that mutant KRAS depletion by AUTACE depends on KPY and is enabled by LIFU‐triggered release.

Given that AUTACE activates CD8^+^ T cells and degrades mutant KRAS, we assessed the cytotoxic activity mediated by AUTACE in cooperation with CD8^+^ T cells. Luciferase‐expressing PANC‐1 and MIA PaCa‐2 cells were co‐cultured with human CD8^+^ T cells in the presence of various nanoparticles. The AUTACE + T cells + LIFU group produced the greatest tumor cell killing compared to all other control groups (Figure 6d,e). In addition, fluorescence imaging confirmed the lowest number of residual tumor cells in the AUTACE + T cells + LIFU group compared to all controls (Figure 6f). Notably, under matched T cell–to–tumor cell ratios, AUTACE + T cells + LIFU induced significantly greater tumor cell killing than PFP@TCE + T cells + LIFU (Figure S11a,b), supporting a cooperative antitumor effect arising from KRAS degradation and CD8^+^ T‐cell activation.

Finally, the stability of AUTACE was evaluated. AUTACE maintained a stable size distribution in PBS (Figure S12a), the cumulative spontaneous release of KPY from AUTACE did not exceed 30% over 10 days (Figure S12b). Over the same period, mutant KRAS degradation efficiency, tumor cell binding, and CD8^+^ T‐cell activation showed no significant changes (Figure S12c–e), thus demonstrating that its functional stability was preserved.

When taken together, these results demonstrate that AUTACE recognizes TP53/KRAS co‐mutant tumor cells via membrane‐displayed TCRs, induces KRAS degradation through the LIFU‐triggered release of KPY, and engages CD8^+^ T cells through surface‐anchored anti‐CD3, thereby achieving antitumor effects in these co‐mutant tumor cells.

### 2.7 Biodistribution of AUTACE

**FIGURE 7:**
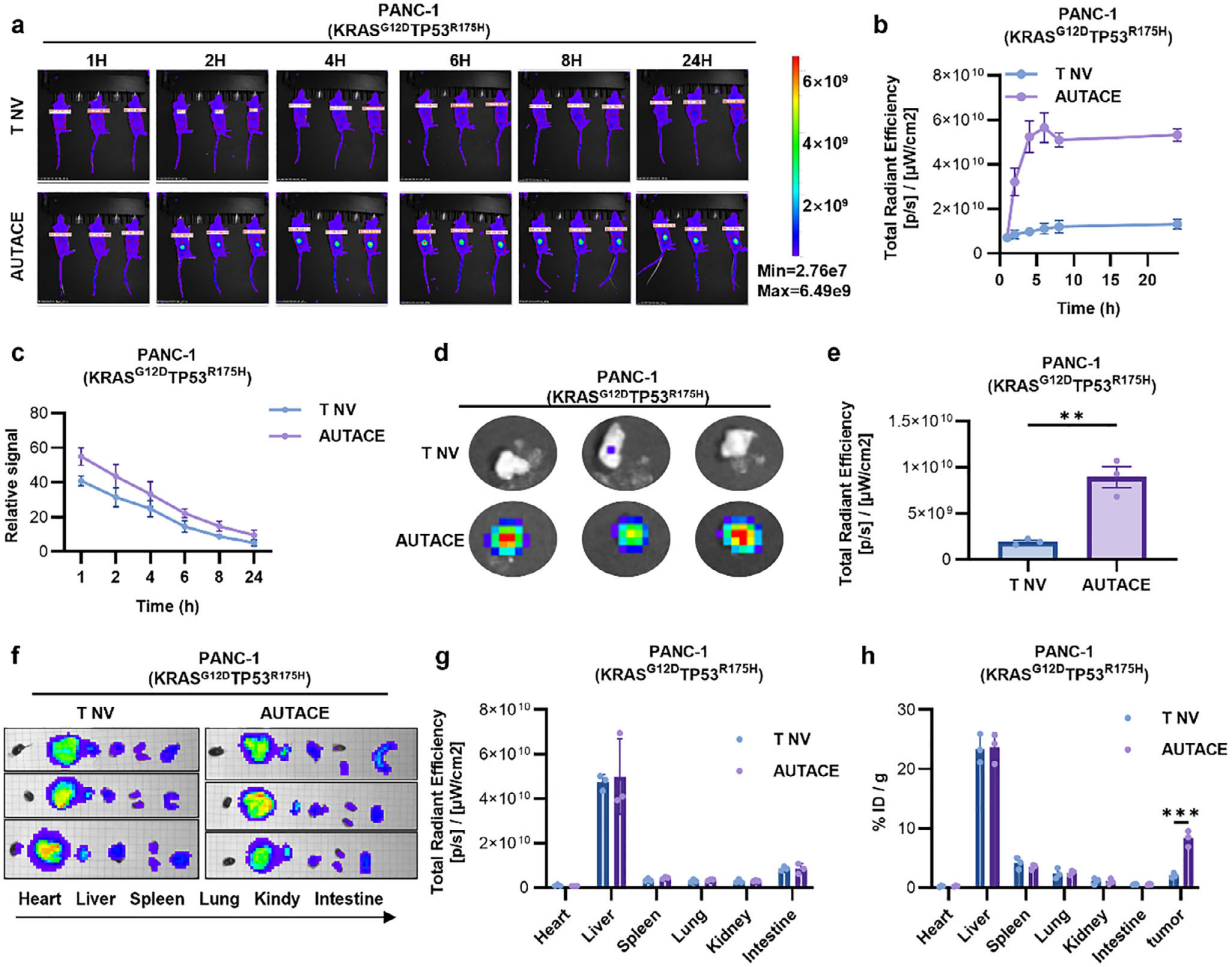
Biodistribution of AUTACE in PANC‐1 tumor‐bearing mice. (a,b) Whole‐body fluorescence imaging (a) and quantitative analysis of tumor‐site fluorescence intensity (b) at the indicated time points after tail‐vein injection of DiD‐labeled T NV or AUTACE (50 mg/kg) in NOG mice bearing PANC‐1 tumors (*n* = 3 per group). (c) Quantification of blood DiD fluorescence signals after injection of DiD‐labeled T NV or AUTACE (*n* = 3). (d,e) Ex vivo fluorescence images of resected PANC‐1 tumors at 48 h after treatment (d) and corresponding quantitative analysis of fluorescence intensity (e) (*n* = 3 per group). (f,g) Ex vivo fluorescence images of resected organs at 48 h after treatment (f) and corresponding quantitative analysis of fluorescence intensity (g) (*n* = 3 per group). (h) Quantitative analysis of the biodistribution of T NV and AUTACE in various organs at 48 h after injection, expressed as percentage of injected dose per gram of tissue (%ID/g) (*n* = 3 per group). T NV: liposomes fused with plasma membranes from T cells lacking TCR expression. KPPF@TCR: KPY‐ and PFP‐loaded liposomes fused with TCR‐T cell plasma membranes. AUTACE: KPPF@TCR further conjugated with anti‐CD3 antibodies. Data are presented as mean ± s.d.; statistical significance was assessed by t‐tests (e) and two‐way ANOVA (g, h); ^**^ *p* < 0.01, ^***^ *p* < 0.001.

To evaluate the in vivo tumor‐targeting capability of AUTACE, PANC‐1, or MIA PaC‐2 tumor‐bearing mice were intravenously injected with DiD‐labeled nanoparticles. The tumor‐specific signal of AUTACE was detectable at 2 h and peaked at 6 h post‐injection, whereas the T NV exhibited minimal tumor accumulation (Figure 7a,b; Figure S13a,b). The blood circulation time of AUTACE was longer than that of T NV (Figure 7c). Moreover, *ex vivo* imaging at 48 h revealed a higher tumor fluorescence intensity in the AUTACE group than in the T NV control group (Figure 7d,e; Figure S13c,d), whereas accumulation in non‐tumor organs was similar between the two groups (Figure 7f,g; Figure S13e,f). Consistent with this, biodistribution analysis performed 48 h post‐injection showed that AUTACE exhibited greater tumor accumulation, reaching approximately 8% injected dose per gram (%ID/g) (Figure 7h). Furthermore, the fluorescence imaging of the tumor sections confirmed the accumulation of AUTACE within the tumors (Figure S13g). Collectively, these results indicated that AUTACE has favorable tumor‐targeting properties and can target both TP53 R175H‐ and R248Q‐mutant tumors.

### 2.8 Antitumor Efficacy and Biosafety of AUTACE

**FIGURE 8:**
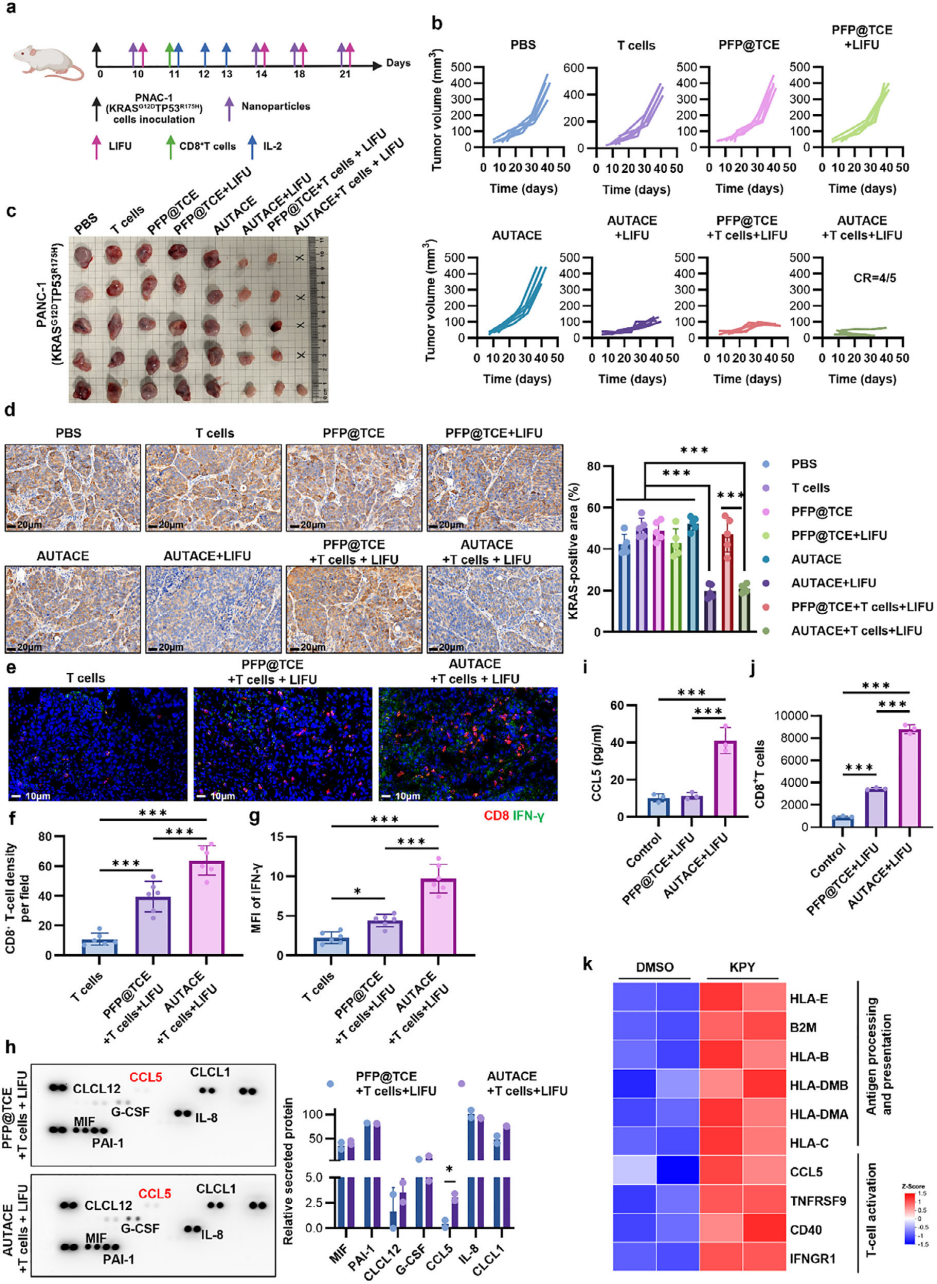
Antitumor effect of AUTACE in PANC‐1 tumor‐bearing mice. (a) Treatment schedule for the in vivo antitumor study, including nanoparticle‐based treatments (50 mg/kg), LIFU irradiation (1 W/cm^2^ for 15 min), IL‐2 administration (2 × 10^5 U), and adoptive transfer of CD8^+^ T cells (1 × 10^7 cells). (b,c) Individual tumor growth curves (b) and tumor images (c) of PANC‐1 tumors after various treatments (n = 5 per group). (d) Representative IHC images of KRAS expression and corresponding quantitative analysis in PANC‐1 tumors at day 21 after tumor inoculation in different treatment groups (*n* = 5 per group). (e–g) Representative immunofluorescence images (e) and quantitative analysis of intratumoral CD8^+^ T‐cell infiltration (f) and IFN‐γ expression (g) in PANC‐1 tumors at day 21 after tumor inoculation in the indicated treatment groups (*n* = 5 per group). (h) Cytokine array analysis of PANC‐1 tumor lysates collected at day 21 after tumor inoculation in the indicated treatment groups (*n* = 2 per group). i) Enzyme‐linked immunosorbent assay quantification of CCL5 in culture supernatants from PANC‐1 cells after the indicated nanoparticle‐based treatments (3 mg/mL), with or without LIFU (1 W/cm^2^ for 5 min) as indicated (*n* = 3 per group). (j) Flow cytometric quantification of CD8^+^ T cells that migrated across transwell inserts in response to the conditioned medium shown in (i) (*n* = 3 per group). (k) Heatmaps of genes related to antigen processing and presentation and T‐cell activation in PANC‐1 cells treated with DMSO or KPY. KPPF@TCR: KPY‐ and PFP‐loaded liposomes fused with TCR‐T cell plasma membranes. PFP@TCE: PFP‐loaded liposomes fused with TCR‐T cell plasma membranes and conjugated with anti‐CD3 antibodies. AUTACE: KPPF@TCR further conjugated with anti‐CD3 antibodies. Data are presented as mean ± s.d.; statistical significance was assessed by one‐way ANOVA (d,f,g,i,j) and two‐way ANOVA (h); ^*^ *p* < 0.05, ^***^ *p* < 0.001.

The in vivo antitumor efficacy of AUTACE was assessed using PANC‐1 and MIA PaCa‐2 subcutaneous xenograft models. Mice were divided into eight treatment groups and monitored over time (Figure 8a). Compared to the PBS, PFP@TCE, AUTACE, PFP@TCE+LIFU, and T cells groups, AUTACE+LIFU significantly suppressed PANC‐1 tumor growth (Figure 8b,c). Notably, the addition of adoptive CD8^+^ T‐cell transfer (AUTACE + T cells+ LIFU) further reduced the tumor burden, resulting in complete regression in four of five mice (Figure 8b,c). Consistent with the PANC‐1 results, a similar antitumor trend was observed in the MIA PaCa‐2 subcutaneous xenograft model (Figure S14a–c). As expected, tumor tissues from the AUTACE+LIFU and AUTACE+T cells+LIFU groups exhibited reduced KRAS protein expression, as assessed by immunohistochemistry (Figure 8d).

Moreover, the performed immunofluorescence analysis revealed a significant increase in tumor‐infiltrating CD8^+^ T cells and elevated IFN‐γ levels in the AUTACE+T cells+LIFU group compared to the T cells and PFP@TCE+T cells+LIFU control groups, suggesting that KRAS downregulation promotes CD8^+^ T‐cell recruitment and activation (Figure 8e–g). Given the established role of KRAS signaling in cytokine regulation [43, 44], we profiled tumor cytokines and found that AUTACE+T cells+LIFU treatment specifically upregulated CCL5, a key chemokine for CD8^+^ T cell recruitment (Figure 8h). This in vivo finding was corroborated in vitro: AUTACE + LIFU induced higher CCL5 secretion from PANC‐1 cells than PFP@TCE + LIFU (Figure 8i). Functionally, migration significantly increased when the CD8^+^T cells were cultured in conditioned medium from AUTACE+LIFU‐treated cells (Figure 8j). In addition, when we silenced CCL5 in PANC‐1 cells using siRNA (Figure S14d), AUTACE + LIFU treatment resulted in significantly lower CCL5 secretion in siCCL5 cells than in siCtrl cells (Figure S14e), accompanied by a concomitant decrease in CD8^+^ T‐cell migration toward the conditioned medium (Figure S14f). RNA‐seq analysis of KPY‐treated cells further supported this mechanism, showing the upregulated expression of CCL5 alongside genes involved in antigen presentation and T cell activation (Figure 8k).

Finally, we evaluated the potential off‐target effects of the AUTACE therapy. The mice in all of the groups maintained stable hematological parameters (Figure S15a). Alanine aminotransferase (ALT), a serum marker of hepatic injury, did not differ among the treatment groups (Figure S15b). Similarly, the serum creatine kinase (CK; cardiac injury marker) and creatinine (CREA; renal function marker) levels showed no between‐group differences (Figure S15c,d). Finally, histological analyses of the heart, liver, spleen, lungs, kidneys, and intestines revealed no treatment‐related morphological lesions (Figure S15e and Table S2). In addition, leukocyte infiltration in these tissues did not increase after treatment, indicating that AUTACE did not elicit tissue inflammatory responses under the conditions tested (Figure S15e and Table S2). These findings indicated that AUTACE was well tolerated following systemic administration. Taken together, these above results demonstrate that AUTACE exhibits a favorable safety profile and potent antitumor activity against KRAS/TP53 co‐mutant tumors.

## 3 Discussion

Genetic alterations are major drivers of tumor initiation and progression, and co‐mutations in the oncogene KRAS and the tumor suppressor TP53 define a clinically important subset of highly lethal malignancies [1, 45]. Previous studies have shown that KRAS/TP53 co‐mutations establish a cooperative oncogenic network by sustaining proliferative and survival signaling, promoting immune evasion, and rewiring tumor metabolism, thereby conferring pronounced aggressiveness and therapeutic resistance [19, 46, 47, 48]. Consequently, therapies targeting a single pathway or node are often insufficient to achieve durable responses in this setting. In this study, we developed AUTACE, a bifunctional nanoplatform that couples the selective degradation of mutant KRAS with the engagement of CD8^+^ T cells. By simultaneously suppressing KRAS signaling and mobilizing adaptive antitumor immunity, AUTACE effectively controlled KRAS/TP53 co‐mutant tumors.

We first synthesized KPY by conjugating the KRAS‐binding macrocyclic peptide KRpep‐2d to the autophagy ligand YOK‐1304. KPY selectively degrades multiple KRAS mutants via p62‐dependent autophagy. Similar to the recently reported pan‐KRAS degraders, KPY displays activity against several mutant isoforms, including G12D, G12V, and G12C. However, our data indicate that effective degradation of KRAS in cells generally requires KPY concentrations above 10 µM, which are higher than those reported for some existing small‐molecule KRAS degraders [24, 25]. This observation suggests that, while KPY is mechanistically effective, further chemical optimization will be required to improve its potency.

Cell membrane‐derived nanoparticles have emerged as a promising therapeutic strategy because membrane proteins and receptors can endow nanocarriers with intrinsic tumor‐targeting properties [49]. For example, several groups in the past have generated bispecific nanoparticles by coating nanocarriers with tumor cells and immune cell membranes, thereby enabling targeted drug delivery and the activation of antitumor immune responses [50, 51, 52, 53]. Unlike these approaches, our study did not rely solely on native receptors on cell membranes. Instead, we engineered TCR‐T cells expressing TP53 R175H‐ and R248Q‐specific TCRs and used their plasma membranes to generate nanovesicles, enabling AUTACE to display two TCRs that recognize different tumor antigens and thereby co‐target TP53‐mutant tumor subclones.

Our results provide preliminary evidence that AUTACE not only exerts direct cytotoxic effects through KRAS degradation and CD8^+^ T cell redirection but is also associated with increased tumor‐derived CCL5 expression and enhanced antigen presentation. These observations are consistent with previous reports showing that inhibition of KRAS signaling promotes the infiltration and activation of cytotoxic T cells and augments antigen presentation within the tumor microenvironment [43, 44]. However, although increased CCL5 expression was observed following mutant KRAS degradation, the upstream signaling pathways linking KRAS loss to chemokine induction were not directly interrogated in the present study. Elucidation of the signaling intermediates downstream of KRAS that regulate CCL5 expression will be an important direction for future studies.

In conclusion, our data demonstrate the feasibility of coupling targeted degradation of mutant KRAS with CD8^+^ T cell engagement within a single nanoplatform. AUTACE provides a proof of concept for integrating intracellular oncogene elimination with adaptive immune activation in KRAS/TP53 co‐mutant tumors.

## 4 Limitations of the Study

Here, we employed an immunodeficient mouse model with adoptive transfer of human CD8^+^ T cells to assess the in vivo antitumor activity of AUTACE. While this model enabled focused evaluation of TCR‐dependent tumor recognition and CD8^+^ T cell–mediated cytotoxicity, it precluded comprehensive analysis of AUTACE‐induced remodeling of the tumor immune microenvironment and limited assessment of its effects on endogenous immunity, including CD4^+^ T cells and innate immune components. In addition, the applicability of AUTACE may be limited by its dependence on mutant TP53‐specific TCR recognition, which requires both the presence of the relevant TP53 mutation in tumor cells and the expression of the matched HLA allele in patients. Consequently, this strategy may only be applicable to a subset of tumors and patients. Moreover, AUTACE activates CD8^+^ T cells via surface‐anchored anti‐CD3 antibodies. Although robust T‐cell activation was observed, potential immune‐related adverse effects, such as T‐cell exhaustion, activation‐induced cell death, and cytokine‐associated toxicity, were not evaluated in the present study. Future studies employing humanized mouse models will be required to delineate the impact of AUTACE on diverse immune cell subsets and intercellular immune networks.

## Funding

Chongqing Technological Innovation and Application Development (CSTB2024TIAD‐KJFZMSX0023); Natural Science Foundation of Chongqing (CSTB2023NSCQ‐BHX0148); China Postdoctoral Science Foundation (2025M781438); Chongqing Science and Health Joint Medical Research Project (2026QNXM021); Scientific and Technological Research Project of the Chongqing Municipal Education Commission (KJQN202500410, KJZD‐K202500410).

## Conflicts of Interest

The authors declare no conflicts of interest.

## Ethical Statement

Mouse studies were approved by Institutional Animal Care and Use of Chongqing Medical University. All experiments using human T cells were approved by the Ethics Committee of The First Affiliated Hospital of Chongqing Medical University. All individuals signed an informed consent form.

## Supporting information




**Supporting File**: advs75219‐sup‐0001‐SuppMat.doc.

**Supporting File**: advs75219‐sup‐0002‐TableS1.docx.

**Supporting File**: advs75219‐sup‐0003‐TableS2.docx.

## Data Availability

The data that support the findings of this study are available from the corresponding author upon reasonable request.
