# Supplementary material for: The AUTACE That Degrades KRAS and Engages CD8+ T Cells for the Treatment of KRAS/TP53 Co‐Mutant Tumors
Source: Adv Sci (Weinh). 2026 Apr 7:e18455. Online ahead of print. doi: 10.1002/advs.202518455 (PMC13334621; doi:10.1002/advs.202518455)
Supplement: Supplementary file 1 — Supporting File: advs75219‐sup‐0001‐SuppMat.doc. [file ADVS-9999-e18455-s003.doc]

**Supporting Information**

**1. Methods and materials**

**KPY synthesis:** KRB and KPY were custom-synthesized by Xi’an Ruixi Biological Technology, and the detailed synthetic procedures are described below.

Synthesis and characterization of KRB: Starting from the macrocyclic peptide KRpep-2d, which recognizes KRAS mutations, lysine residues were introduced at both the N- and C-termini, and the C-terminal lysine was conjugated to biotin to afford the peptide KRB. The purity and molecular weight of KRB were confirmed using analytical high-performance liquid chromatography (Waters 2695) and mass spectrometry (Waters ZQ2000).

Synthesis of YOK–NH₂: YOK-1304 (10 mg, MCE, Cat. No. HY-161739) was dissolved in anhydrous *N, *N-dimethylformamide (DMF, 1 mL). N-BOC-6-aminohexanoic acid (1.1 equiv), 1-ethyl-3-(3-dimethylaminopropyl) carbodiimide hydrochloride (EDC, 2.0 equiv), and 4-dimethylaminopyridine (DMAP, 1.5 equiv) were added with stirring, and the reaction mixture was allowed to proceed at room temperature for 12 h. The solvent was removed under reduced pressure, and the residue was dispersed in dichloromethane (DCM, CH₂Cl₂, 2 mL). Trifluoroacetic acid (TFA, 0.5 mL) was then added, and the mixture was stirred at room temperature for 0.5 h to remove the BOC protecting group. The organic phase was washed three times with water, dried over anhydrous sodium sulfate, filtered, and concentrated. The resulting solution was slowly added into a large volume of ice-cold diethyl ether to precipitate the product. The precipitate was collected by filtration and dried via vacuum to obtain YOK–NH₂.

Synthesis of YOK–PEG2000–COOH: YOK–NH₂ (5 mg) was dissolved in DCM (1 mL), followed by the addition of N-hydroxysuccinimide–poly (ethylene glycol) 2000–carboxylic acid (NHS–PEG2000–COOH, 1.1 equiv) and triethylamine (TEA, 2.0 equiv). The reaction mixture was then stirred at room temperature for 4 h. The organic phase was washed three times with water, dried over anhydrous sodium sulfate, filtered, and concentrated under reduced pressure. The residue was precipitated by slow addition of the solution to ice-cold diethyl ether. The precipitate was collected via filtration and dried under vacuum to obtain YOK–PEG2000–COOH.

Synthesis of KPY: YOK–PEG2000–COOH (5 mg) was dissolved in anhydrous DMF (1 mL), and EDC (2.0 equiv) and N-hydroxysuccinimide (NHS, 2.0 equiv) were added. The mixture was stirred at room temperature for 12 h to activate the terminal carboxyl group. The solvent was removed under reduced pressure, and the residue was dissolved in DCM (1 mL), washed three times with water, dried over anhydrous sodium sulfate, and then concentrated. The crude activated ester was redissolved in DMF (1 mL), followed by the addition of the KRB peptide (1.1 equiv) and TEA (3.0 equiv). After stirring at room temperature for 12 h, the reaction mixture was transferred to a dialysis bag (molecular weight cut-off, 3.5 kDa) and dialyzed against deionized water for 12 h. The dialysate was lyophilized, and the resulting solid was precipitated with ice-cold diethyl ether and dried under vacuum to yield KPY. The molecular weight and chemical structure of KPY were confirmed using mass spectrometry and proton nuclear magnetic resonance (Bruker 400 MHz spectrometer with an autosampler).

**Molecular docking:** KRAS G12D was used as the receptor and KPY as the ligand. The receptor structure was predicted using AlphaFold3 and an optimal model was selected. For AutoDock Vina, the receptor was prepared by removing ligands and water, completing missing residues, adding hydrogen atoms, assigning Gasteiger charges, and converting to PDBQT; the ligand was prepared by building a 3D structure, adding hydrogen atoms, setting ionization (protonation) states at physiological pH, performing energy minimization, and converting to PDBQT. The docked poses were filtered using binding energies and key interactions, and the selected complexes were visualized using PyMOL.

**Circular dichroism spectroscopy:** KRAS G12D (0.42 μM, 1 Ml, GenScript, Cat. No. Z03598), KRAS G12C (0.42 μM, 1 mL, GenScript, Cat. No. Z03597), KRAS G12V (0.45 μM, 1 mL, Beyotime, Cat. No. P2413), and wild-type KRAS (0.45 μM, 1 mL, Beyotime, Cat. No. P2407) were each incubated with KPY (5 mg/mL, 20 μL), KRB (1 mg/mL, 100 μL), or YOK-1304 (10 mM, 23 μL) at 37 °C for 30 min. Circular dichroism spectra of the protein solutions were recorded using a Jasco spectropolarimeter.

**Biolayer interferometry analysis:** Biolayer interferometry measurements were performed using an Octet R2 instrument (ForteBio). KRB or KPY were immobilized at 10 μg/mL onto streptavidin-coated biosensors to a loading response of ~1.5 nm. Before performing measurements, the sensors (Sartorius, Cat. No. 18-5019) were pre-equilibrated in kinetic buffer (PBS containing 0.1% Tween-20 and 5% BSA) for 120 s to block nonspecific sites and establish a stable baseline. For kinetic analyses, recombinant proteins were prepared by 2-fold serial dilution in kinetic buffer to final concentrations of 50, 25, 12.5, 6.25, and 3.125 nM for KRAS G12D, or 100, 50, 25, 12.5, and 6.25 nM for KRAS G12C, KRAS G12V, HRAS (Biolegend, Cat. No. P2403), and wild-type KRAS. The association was recorded for 120 s, followed by a 240-s dissociation in buffer. Data were processed using the FortéBio Data Analysis software with reference subtraction and a global 1:1 binding model to calculate the association rate constant (k_on_), dissociation rate constant (k_dis_), and equilibrium dissociation constant (K_D_).

**Cellular uptake of KPY:** PANC-1, MIA PaCa-2, SK-CO-1, and HCC827 cells were incubated separately with PE-labeled KPY at 0, 1.25, 2.5, 5, and 10 μM for 12 h, and intracellular KPY was quantified by flow cytometry.

**Immunofluorescence:** Tumor cells were seeded on glass coverslips, allowed to adhere overnight, and then treated as indicated. For colocalization of KPY with KRAS, the cells were incubated with KPY (20 μM) for 12 h, washed with PBS, and incubated overnight at 4 °C with an anti-KRAS (Proteintech, Cat. No. 12063-1-AP) primary antibody; For colocalization of LC3 with p62, tumor cells were treated for 12 h with KRB, KPY, or YOK-1304 (each at 20 μM), then incubated with anti-LC3 (Cell Signaling Technology, Cat. No. 12741S) and anti-p62 (Proteintech, Cat. No. 66184-1-Ig) primary antibodies; For other colocalization analyses, including LC3 with KRAS, p62 with KRAS, and KRAS with LAMP1, cells were treated with KPY (20 μM, 12 h) and incubated with the corresponding primary antibody pairs: LC3 (Proteintech, Cat. No. 66139-3-Ig) with KRAS (Proteintech, Cat. No. 12063-1-AP); p62 (Proteintech, Cat. No. 66184-1-Ig) with KRAS (Proteintech, Cat. No. 12063-1-AP); or KRAS (Proteintech, Cat. No. 12063-1-AP) with LAMP1 (Proteintech, Cat. No. 65051-1-Ig); After primary antibody incubation, cells were incubated for 2 h at room temperature with the appropriate fluorophore-conjugated secondary antibodies or PE–streptavidin, including FITC- or Cy3-conjugated AffiniPure goat anti-rabbit IgG (H+L) and goat anti-mouse IgG (H+L) (Proteintech, Cat. No. SA00003-2, SA00009-2, SA00003-1, and SA00009-1), as well as PE–streptavidin (BioLegend, Cat. No. 405203). Nuclei were counterstained with DAPI (Beyotime, Cat. No. C1006) for 30 min. For autophagic flux analysis using the mCherry–EGFP–LC3 reporter, PANC-1 cells stably expressing mCherry–EGFP–LC3 (BioLegend, Cat. No. C3022) were treated with KPY (20 μM) or KPY in combination with hydroxychloroquine (HCQ, 10 μM; Selleck, Cat. No. E4824) for 12 h, followed by nuclear counterstaining with DAPI. Images were acquired using a laser-scanning confocal microscope (Andor Dragonfly 200). For tissue immunofluorescence analysis of CD3 and IFN-γ, OCT-embedded tissues were cryosectioned (8 μm) and stained with anti-CD3 (Servicebio, Cat. No. GB12014) and anti–IFN-γ (Affinity, Cat. No. DF6045) primary antibodies, followed by species-appropriate fluorescent secondary antibodies. Nuclei were counterstained with DAPI before imaging.

**Western blot.** Tumor cells were lysed, and total protein was extracted. Protein concentrations were determined using bicinchoninic acid (BCA, Beyotime, Cat. No. P0009). Equal amounts of protein were denatured in Laemmli buffer, resolved on 12% SDS–polyacrylamide gels at 150 V for ~50 min, and transferred onto PVDF membranes. Membranes were blocked in 5% non-fat milk in TBST for 2 h at room temperature, incubated overnight at 4 °C with primary antibodies against KRAS (Proteintech, Cat. No. 12063-1-AP), LC3 (Cell Signaling Technology, Cat. No. 12741S), p62 (Proteintech, Cat. No. 66184-1-Ig), β-actin (Proteintech, Cat. No. 66009-1-Ig), or His-tag (Affinity, Cat. No. T0009). After washing, membranes were incubated with HRP-conjugated secondary antibodies (Proteintech, Cat. No. RGAR011 and RGAM011) for 2 h at room temperature. Protein signals were visualized using enhanced chemiluminescence (ECL, Beyotime, Cat. No. P0018M).

**In vitro cell proliferation and apoptosis assays**. Tumor cells (1 × 10^4 per well) were seeded in 96-well plates and allowed to adhere overnight. For the cell proliferation assays, cells were treated with KPY at 3.125, 6.25, 12.5, 25, or 50 μM for 48 h. For apoptosis assays, cells were treated with KPY at 20 μM for 48 h. Proliferation was quantified using a CCK-8 kit (Beyotime, Cat. No. C0040) according to the manufacturer’s instructions, and absorbance was read at 450 nm on a microplate reader. Apoptosis was assessed using an apoptosis detection kit (Biolegend, Cat. No. 640930) following the manufacturer’s protocol and analyzed by flow cytometry.

**RNA-seq and metabolomics analyses:** PANC-1 cells were treated with DMSO or KPY (20 μM) for 24 h, then harvested. RNA-seq was performed by Majorbio Pharm Tech Co., Ltd. (Shanghai, China) and metabolomics profiling was conducted by Shanghai Applied Protein Technology Co., Ltd. (Shanghai, China).

**Plasma stability of KPY and KRB:** KPY and KRB (10 µg/mL; 50 µL each) were added separately to mouse plasma (5 mL) and incubated at 37 °C for 0, 2, 6, 12, and 24 h. At each time point, 200 µL of plasma was withdrawn and mixed with 400 µL protein-precipitation solvent (80% acetonitrile, 19.5% acetone, 0.5% formic acid). Samples were vortexed for 15 s, sonicated in an ice-water bath for 2 min, and shaken at 4 °C for 10 min, then centrifuged at 15,000 g and 4 °C for 15 min. The supernatants were subjected to UHPLC separation on a Vanquish Flex system, and the analytes were detected by high-resolution mass spectrometry using a Q-Exactive HF-X instrument.

**CD8^+^ T cell priming:** Peripheral blood was collected from healthy adult volunteers with informed consent. PBMCs were isolated from anticoagulated whole blood using Lymphoprep (STEMCELL Technologies, Cat. No.18061) according to the manufacturer’s instructions. CD8⁺ T cells were then purified from PBMCs using the EasySep™ Human CD8^+^ T Cell Enrichment Kit (STEMCELL Technologies, Cat. No.19053) according to the standard magnet-assisted protocol.

**CRISPR/Cas9 ribonucleoprotein electroporation:** sgp62 (GCGCCTCCTGAGCACACGGT) or the sgTRAC (AGAGTCTCTCAGCTGGTACA) and sgTRBC (GGAGAATGACGAGTGGACCC), as well as non-targeting control sgRNA (ATCTGAGCGTTTTCGGCCGC), were mixed with recombinant Cas9 protein (GenScript, Z03621-1) in order to assemble ribonucleoprotein (RNP) complexes at room temperature. PANC-1 cells and pre-activated human CD8⁺ T cells were harvested, washed, and resuspended in Lonza SE or P3 Nucleofector solutions (Lonza, Cat. No. V4XC-1032 and V4XP-3032), respectively. Cells were then electroporated with Cas9–sgRNA RNP complexes using a Lonza 4D-Nucleofector, according to programs optimized for each cell type. After electroporation, the cells were immediately transferred into pre-warmed complete medium and cultured for recovery.

**Plasmid and Lentivirus vector construction and cellular transduction:** Plasmids encoding TP53 R175H–specific TCR, TP53 R248Q–specific TCR, TP53 R175H-HA, TP53 R248Q-HA, and His-KRAS were synthesized by Tsingke and subcloned into the lentiviral transfer vector pWPXL (Addgene). Lentiviral particles were produced by co-transfecting Lenti-X 293T cells with the pWPXL constructs, packaging plasmid psPAX2, and the envelope plasmid pMD2.G using the Xfect Transfection Reagent (Takara, Cat. No.631318). CD8⁺ T cells were activated with anti-CD3/CD28 Dynabeads (Thermo Fisher Scientific, Cat. No.11132D) for 24 h and then transduced with lentiviruses encoding TP53 R175H- and R248Q-specific TCRs. PANC-1 and MIA PaCa-2 cells were separately transduced with TP53 R175H-HA or TP53 R248Q-HA lentiviruses to establish stable cell lines. PANC-1 cells were transduced with His-KRAS lentiviruses to establish a stable cell line.

**siRNA-mediated knockdown of CCL5 in PANC-1 cells:** Cells were transfected with siCCL5 (GCAGAGGATCAAGACAGCA) or a non-targeting control siRNA (TTCTCCGAACGTGTGTCACGT) using Lipofectamine RNAiMAX (Thermo Fisher Scientific, Cat. No.13778150) according to the manufacturer’s instructions. Briefly, siRNA (50 nM) and Lipofectamine RNAiMAX were separately diluted in Opti-MEM (Thermo Fisher Scientific, Cat. No.31985070), combined, and incubated for 15 min at room temperature to form complexes before being added dropwise to the cells. After 6 h, the medium was replaced with fresh complete medium and the cells were further cultured for 48 h.

**Synthesis and characterization of nanoparticles:** Soybean phosphatidylcholine (SPC, 26 mg), cholesterol (1.5 mg), 1,2-distearoyl-sn-glycero-3-phosphoethanolamine–N-[methoxy(polyethylene glycol)-2000] (DSPE–PEG2000, 1 mg), perfluoropentane (PFP, 4.5 mg), and KPY (2 mg) were dissolved in 2 mL chloroform. The organic solvent was removed under reduced pressure to form a thin lipid film on the wall of the round-bottom flask. The lipid film was then hydrated with an aqueous solution containing DSPE–PEG–streptavidin (3 mg) under sonication in an ice-water bath, followed by bath sonication at 180 W and extrusion through a polycarbonate membrane (100 nm pore size) using a mini-extruder to obtain KPY/PFP-loaded liposomes ( KPPF@liposomes). PFP@liposomes and blank liposomes were prepared using the same procedure, omitting KPY and/or PFP as required.

TCR-engineered T cells expressing TP53 R175H- and R248Q-specific T-cell receptors (TCR-T cells) or control T cells lacking endogenous TCRs were collected, washed three times with ice-cold PBS, resuspended in PBS containing a protease inhibitor cocktail, and homogenized. Plasma membranes were isolated by multi-step density gradient centrifugation according to standard protocols to obtain TCR-T cell membranes and control T-cell membranes.

KPPF@liposomes and PFP@liposomes were mixed with TCR-T cell membranes at a mass ratio of 5:1 (liposome: membrane), sonicated in an ice-water bath for 30 s, and incubated at room temperature for 2 h to generate TCR-decorated nanovesicles (KPPF@TCR and PFP@TCR, respectively). Similarly, the fusion of blank liposomes with control T cell membranes yielded T cell nanovesicles (T NV).

To prepare AUTACE and PFP@TCE, 5 mg of KPPF@TCR or PFP@TCR were incubated with 100 μg biotinylated anti-CD3 antibody at 4 °C for 2 h to allow binding through the streptavidin–biotin interaction, yielding AUTACE and PFP@TCE, respectively.

The nanoparticle morphology was examined by transmission electron microscopy. The zeta potential was measured using a NanoBrook 90Plus PALS analyzer. The hydrodynamic size distributions were determined using NanoBrook 90Plus PALS and nanoparticle tracking analysis. The number of anti-CD3 molecules per AUTACE particle was quantified by flow cytometry. The absorbance of KPY at 266 nm was measured using a UV spectrophotometer, and the drug-loading and encapsulation efficiencies were calculated.


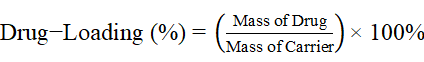


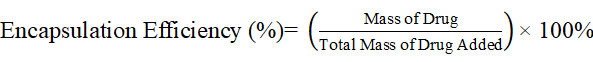


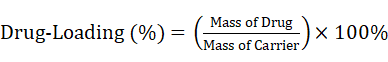


**Drug release assay:** For the drug release studies, AUTACE was diluted in PBS to a concentration of 1 mg/mL. Samples were either exposed to LIFU (1 W/cm² for 5 min) or untreated as controls. The suspension was then placed in a conical flask and incubated at 37 °C in the dark on a shaker (100 rpm). At predefined time points, aliquots were collected and centrifuged to remove nanoparticles, and the supernatants were analyzed by UV–vis spectrophotometry at 266 nm. The cumulative release was calculated from a KPY standard curve.

**Cell binding assay:** For flow cytometry, tumor cells were incubated with DiD (Beyotime, Cat. No. C1039)-labeled nanoparticles (1.2 × 10^10/mL, 100 μL) for 6 h, washed twice with PBS to remove unbound particles, and analyzed by flow cytometry. For confocal microscopy, tumor cells were seeded on glass coverslips and allowed to adhere overnight, then incubated with DiD-labeled nanoparticles (1.2 × 10^10/mL, 100 μL) for 6 h, washed with PBS, and nuclei were counterstained with DAPI for 30 min. Images were acquired using a laser-scanning confocal microscope (Andor Dragonfly 200).

**Cytotoxicity assay:** Luminescence-based killing, 1 × 10^4 luciferase-expressing PANC-1 or MIA PaCa-2 cells were seeded in 96-well plates and incubated overnight. Approximately 3 mg/mL of nanoparticles was added and exposed to LIFU (1 W/cm² for 5 min). CD8⁺ T cells were then added at T cells-to-tumor ratios of 0.5:1, 1:1, 2.5:1, 5:1, and 10:1, and co-cultured for 24 h. AUTACE-mediated cytotoxicity was quantified using the Bio-Lumi™ Firefly Luciferase Reporter Gene Assay Kit (Beyotime, Cat. No. RG042S) with the corresponding Cell Lysis Buffer, following the manufacturer’s instructions.


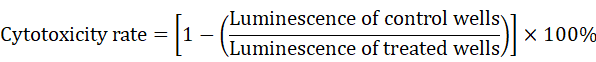


For fluorescence imaging–based killing, 1 × 10^4 DiD-labeled PANC-1 or MIA PaCa-2 cells were seeded in 48-well plates and incubated overnight. Approximately 3 mg/mL of nanoparticles was added and treated with LIFU (1 W/cm² for 5 min). Next, 1 × 10^5 DiO (Beyotime, Cat. No. C1038)-labeled CD8⁺ T cells were added and co-cultured for 24 h. Images were acquired using a fluorescence microscope.

**In vitro CD8⁺ T cell activation assay:** 5 × 10^4 CD8⁺ T cells were seeded in 96-well plates and co-cultured with nanoparticles at the indicated concentrations for 24 h. Cells were harvested, and CD69 expression was quantified by flow cytometry. Cell-free supernatants were collected, and the IFN-γ and GZMB levels were measured by Human IFN-γ ELISA Kit (Beyotime, Cat. No. Pl511) or Human Granzyme B ELISA Kit (Abcam, Cat. No. ab235635), respectively. As a positive control, CD8⁺ T cells were stimulated with 250 nM PMA (MCE, Cat. No. HY-18739) and 250 ng/mL ionomycin (MCE, Cat. No. HY-13434).

**Mice:** NOG mice were purchased from Beijing Vitalstar Biotechnology and maintained at the animal facility of the Chongqing Medical University Experimental Animal Center. NOG mice were subcutaneously inoculated with 2 × 10^7 PANC-1 cells, 1 × 10^7 MIA PaCa-2 cells, or 1 × 10^7 HCC827 cells. When tumor volumes reached approximately 30 mm³, the mice were randomized, and treatments were initiated as indicated.

For in vivo administration, KPY was first dissolved in DMSO (200 mg/mL) and then diluted with PBS before use. For tail-vein administration, NOG mice bearing PANC-1 xenografts received KPY at 5, 10, or 20 mg/kg by intravenous injection (i.v.) every three days for a total of three doses. For intratumoral administration, NOG mice bearing PANC-1 or MIA PaCa-2 xenografts received 5 mg/kg KPY via direct intratumoral injection, whereas NOG mice bearing HCC827 xenografts received 5, 10, or 20 mg/kg KPY intratumorally.

In vivo nanoparticle dosing, LIFU, and adoptive CD8⁺ T cell transfer: For the PANC-1 cohort, NOG mice received tail-vein injections of nanoparticles (50 mg/kg) on days 10, 14, 18, and 21 after tumor implantation. 6 h after each nanoparticle dose, the tumors were exposed to LIFU (1 W/cm² for 15 min). On day 11, mice were given 1 × 10^7 CD8⁺ T cells intravenously, and recombinant human IL-2 (2 × 10^5 U) was administered intraperitoneally (i.p.) on days 11, 12, and 13; For the MIA PaCa-2 cohort, nanoparticles (50 mg/kg, i.v.) were administered on days 5, 9, 13, and 17, followed 6 h later by LIFU (1 W/cm² for 15 min). On day 6, the mice received 1 × 10^7 CD8⁺ T cells intravenously, and IL-2 (2 × 10^5 U, i.p.) was administered on days 6–8.

**In vivo bioluminescence and imaging:** DiD-labeled T NV or AUTACE (50 mg/kg) were administered via tail-vein injection into NOG mice bearing PANC-1 or MIA PaCa-2 tumors. Blood samples were collected 5 min, 1 h, 2 h, 4 h, 6 h, 8 h, and 24 h post-injection. Serum fluorescence was then measured, and the fluorescence intensity at 5 min was set to 100%, with relative fluorescence at subsequent time points used to calculate the circulation profile of the nanoparticles. Whole-body fluorescence was measured at the indicated time points using an IVIS Lumina Series III system (PerkinElmer). After 48 h, the tumors and major organs (heart, liver, spleen, lungs, kidneys, and intestines) were collected and then subjected to additional IVIS imaging to analyze the luminescence signals from these organs. For quantitative analysis of nanoparticles in tissues, the excised tumors and organs were weighed and homogenized, and the fluorescence intensity of the tissue homogenates was measured. Based on a standard curve of nanoparticle fluorescence intensity versus mass, the mass of nanoparticles in each tissue was calculated, and the results were expressed as the percentage of injected dose per gram of the tissue (%ID/g).


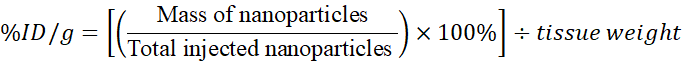


**Cytokine assays:** For cytokine profiling of tumor tissues, tumors were harvested from NOG mice bearing PANC-1 xenografts. Tumor tissues were homogenized in lysis buffer (RD Systems, Cat. No. 895935) supplemented with protease inhibitor cocktail (Sigma, Cat. No. P8340), and cytokines were analyzed using the Proteome Profiler Human Cytokine Array Kit (RD Systems, Cat. No. ARY005B) according to the manufacturer’s instructions. In order to quantify CCL5 in the cell culture supernatants, PANC-1 cells were treated with PFP@TCE or AUTACE (3 mg/mL) and exposed to LIFU (1 W/cm² for 5 min). After 48 h, the culture supernatants were collected and CCL5 levels were measured using a Human CCL5/RANTES Quantikine ELISA Kit (RD Systems, Cat. No. DRN00B) according to the manufacturer’s instructions.

**Transwell assay:** PANC-1 cells were seeded in 6-well plates and incubated overnight. The cells were then treated with PFP@TCE or AUTACE (3 mg/mL) and exposed to LIFU (1 W/cm² for 5 min). After 48 h, the culture supernatants were collected. 5 × 10^5 CD8⁺ T cells were added to the upper chambers of Transwell inserts, and the lower chambers were filled with 600 µL RPMI-1640 or culture supernatants. After 24 h, the cells that had migrated into the lower chambers were harvested and analyzed by flow cytometry.

**2. Statistical analysis**

Statistical analyses were performed using GraphPad Prism 10. Data are presented as mean ± SD. Two-group comparisons were performed using Student’s *t*-tests, and multiple-group comparisons were performed using one-way or two-way ANOVA, as appropriate. Statistical significance was defined as *p < 0.05, ** p < 0.01, and *** p < 0.001.

**3. Supplementary Figures**

**
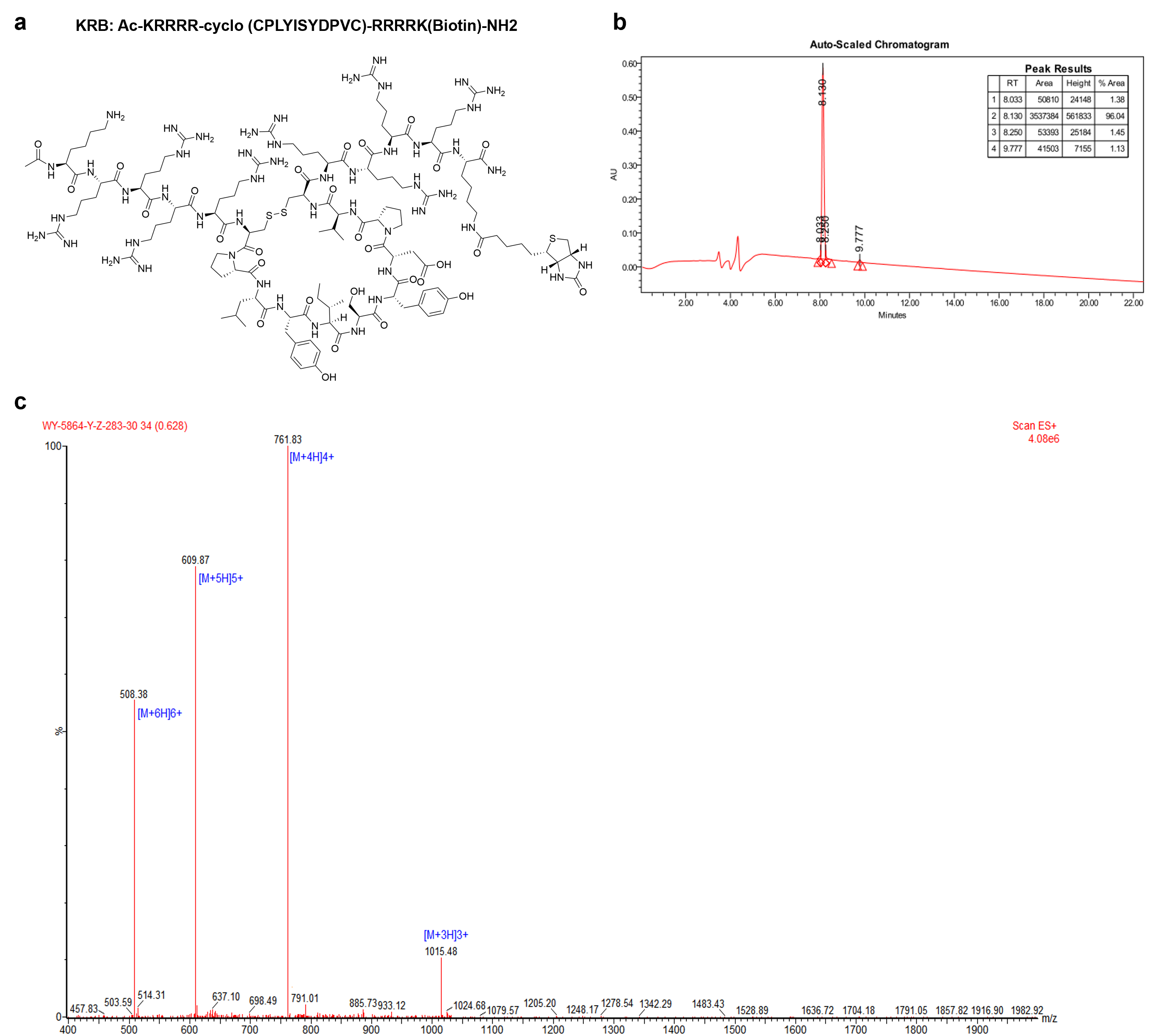
**

**Figure S1.** Characterization of the synthesized KRB peptide. a) Molecular structure of the peptide KRB. b-c) The purity and molecular weight of KRB were confirmed by analytical high-performance liquid chromatography (b) and mass spectrometry (c).


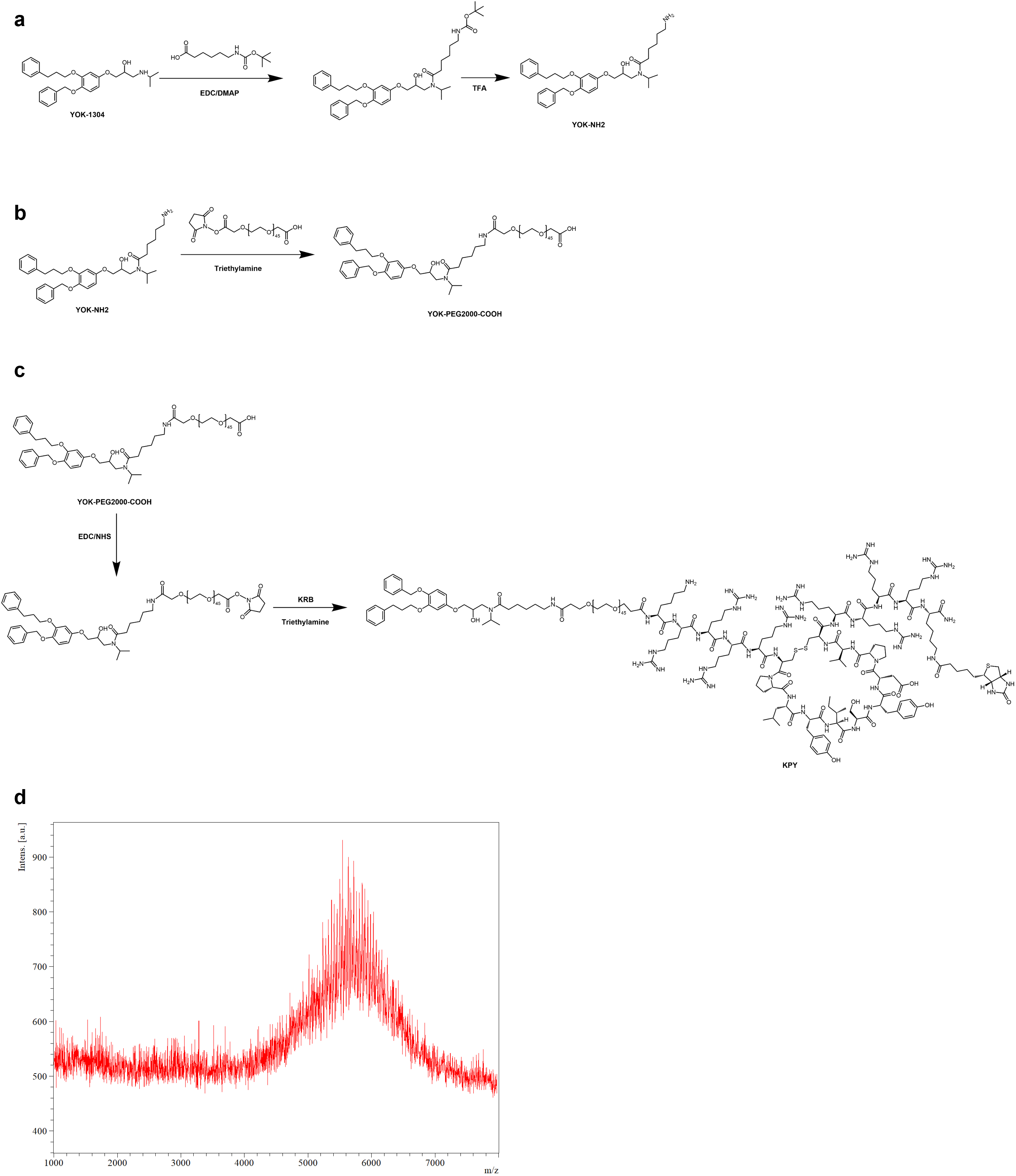


**Figure S2.** Synthesis and characterization of KPY. a-c) Schematic illustration of the synthesis of KPY. d) Mass spectrometry was used to confirm the molecular weight of KPY.


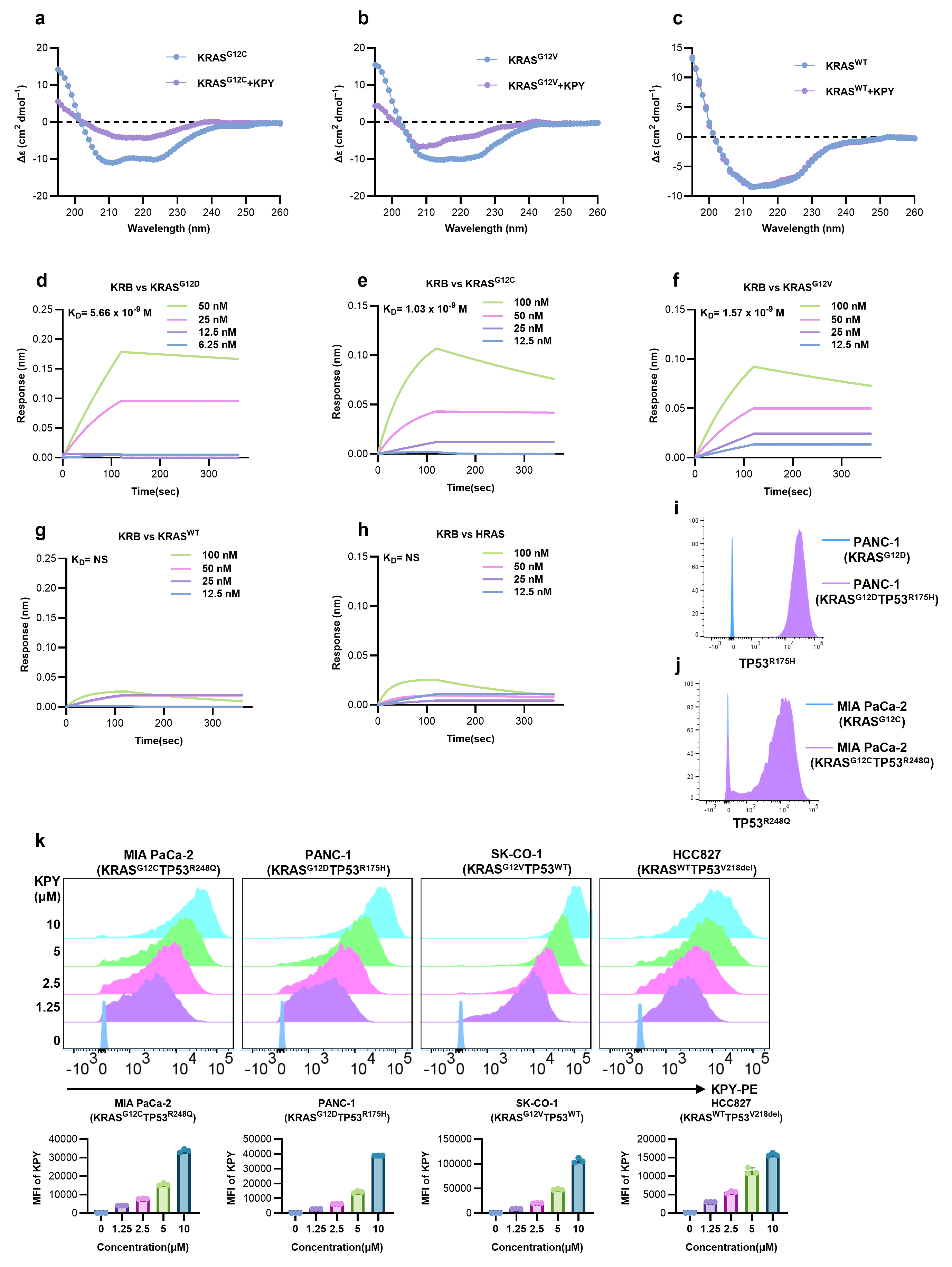


**Figure S3.** KPY and KRB bind mutant KRAS proteins. a-c) Circular dichroism spectra of KRAS G12C, G12D and WT-KRAS mixed with KPY. d-h) Biolayer interferometry was used to measure the affinity of KRB for multiple proteins; representative data from one of three independent experiments are shown. i-j) Flow-cytometric detection of TP53 R175H and R248Q expression in PANC-1 (i) and MIA PaCa-2 (j) cells, respectively. k) Flow-cytometric quantification of KPY uptake in different cell lines (n = 3 per group).


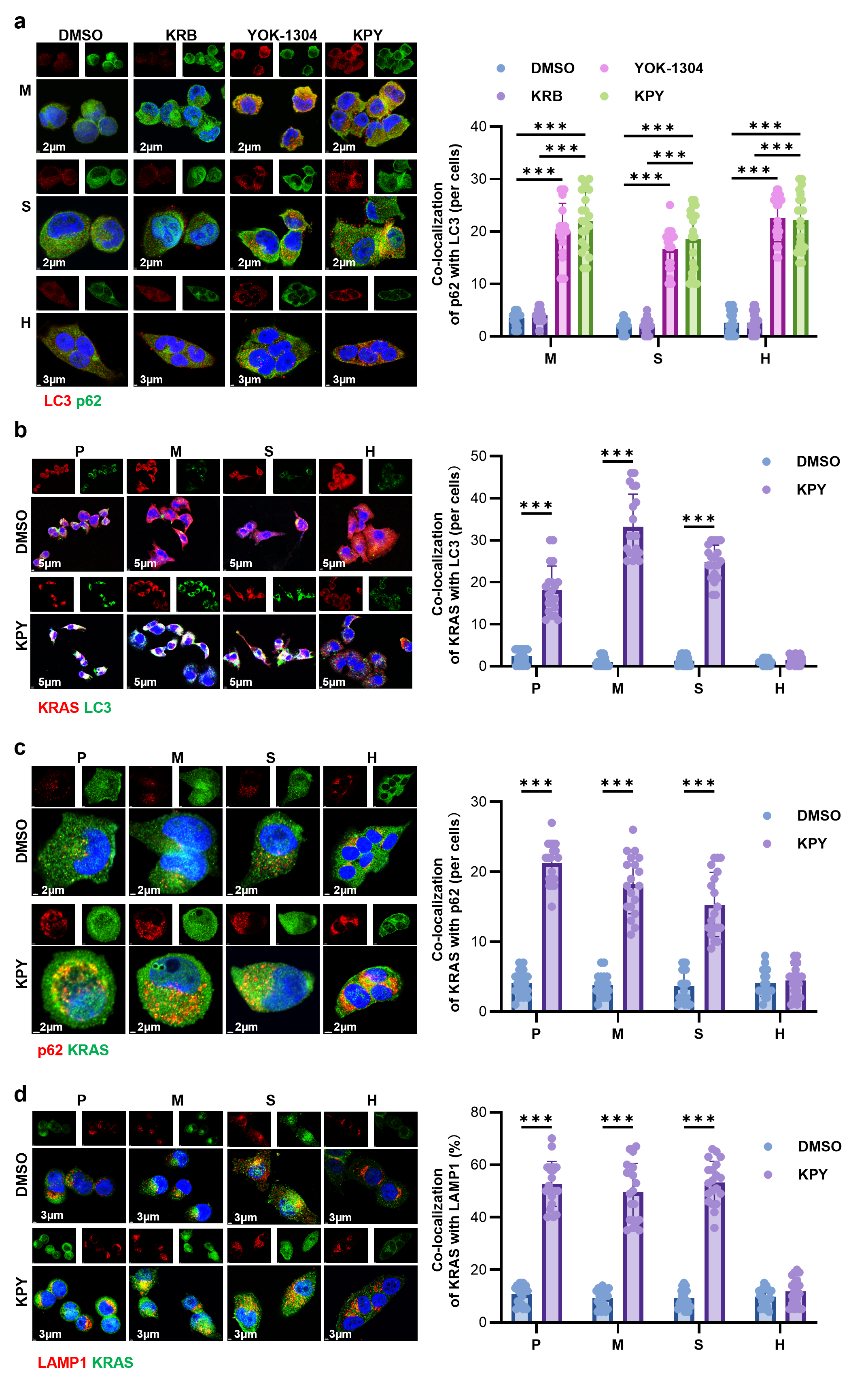


**Figure S4.** Interaction of KPY with cells. a) Representative confocal microscopy images and corresponding quantitative analysis of LC3 and p62 co-localization in different cell lines after treatment with KRB, KPY, or YOK-1304 (20 μM each) 12 h (n = 20 cells per group). b) Representative confocal microscopy images and corresponding quantitative analysis of KRAS and LC3 co-localization in different cell lines treated with DMSO or KPY (20 μM) for 12 h (n = 20 cells per group). c) Representative confocal microscopy images and corresponding quantitative analysis of KRAS and p62 co-localization in different cell lines treated with DMSO or KPY (20 μM) for 12 h (n = 20 cells per group). d) Representative confocal microscopy images and corresponding quantitative analysis of KRAS and LAMP1 co-localization in different cell lines treated with DMSO or KPY (20 μM) for 12 h (n = 20 cells per group). P: PANC-1 cells. M: MIA PaCa-2 cells. S: SK-CO-1 cells. H: HCC827 cells. Data are presented as mean ± s.d.; statistical significance was assessed by two-way ANOVA (a, b, c, d); ***P < 0.001.


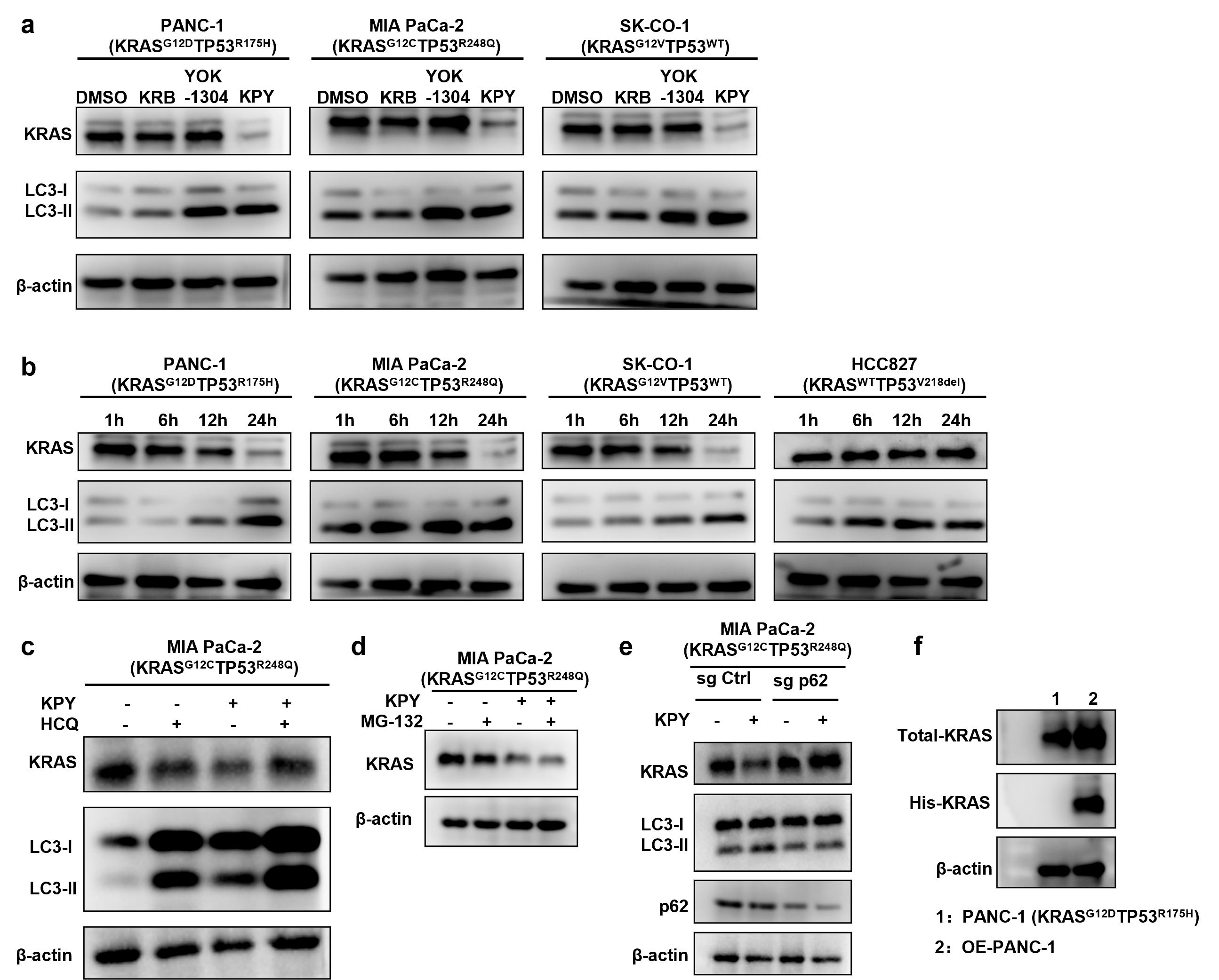


**Figure S5.** KPY induces autophagy-dependent degradation of mutant KRAS. a) Western blot analysis of KRAS, LC3, and β-actin in different cell lines after treatment with KRB, KPY, or YOK-1304 (20 μM each) for 24 h. b) Western blot analysis of KRAS, LC3, and β-actin in different cell lines after the KPY (20 μM) treatments at different time points. c) Western blot analysis of KRAS, LC3, and β-actin in MIA PaCa-2 cells treated with KPY (20 μM), HCQ (10 μM), or the combination for 24 h. d) Western blot analysis of KRAS and β-actin in MIA PaCa-2 cells treated with KPY (20 μM), MG-132 (1 μM), or the combination for 24 h. e) Western blot analysis of KRAS, LC3, p62, and β-actin in MIA PaCa-2 cells with sgRNA-mediated knockdown of p62 treated with KPY (20 μM) for 24 h. f) Western blot analysis of KRAS and His-KRAS in PANC-1 cells overexpressing His-tagged WT KRAS (OE-PANC-1). For all western blot data, representative images from one of three independent experiments are shown.

**
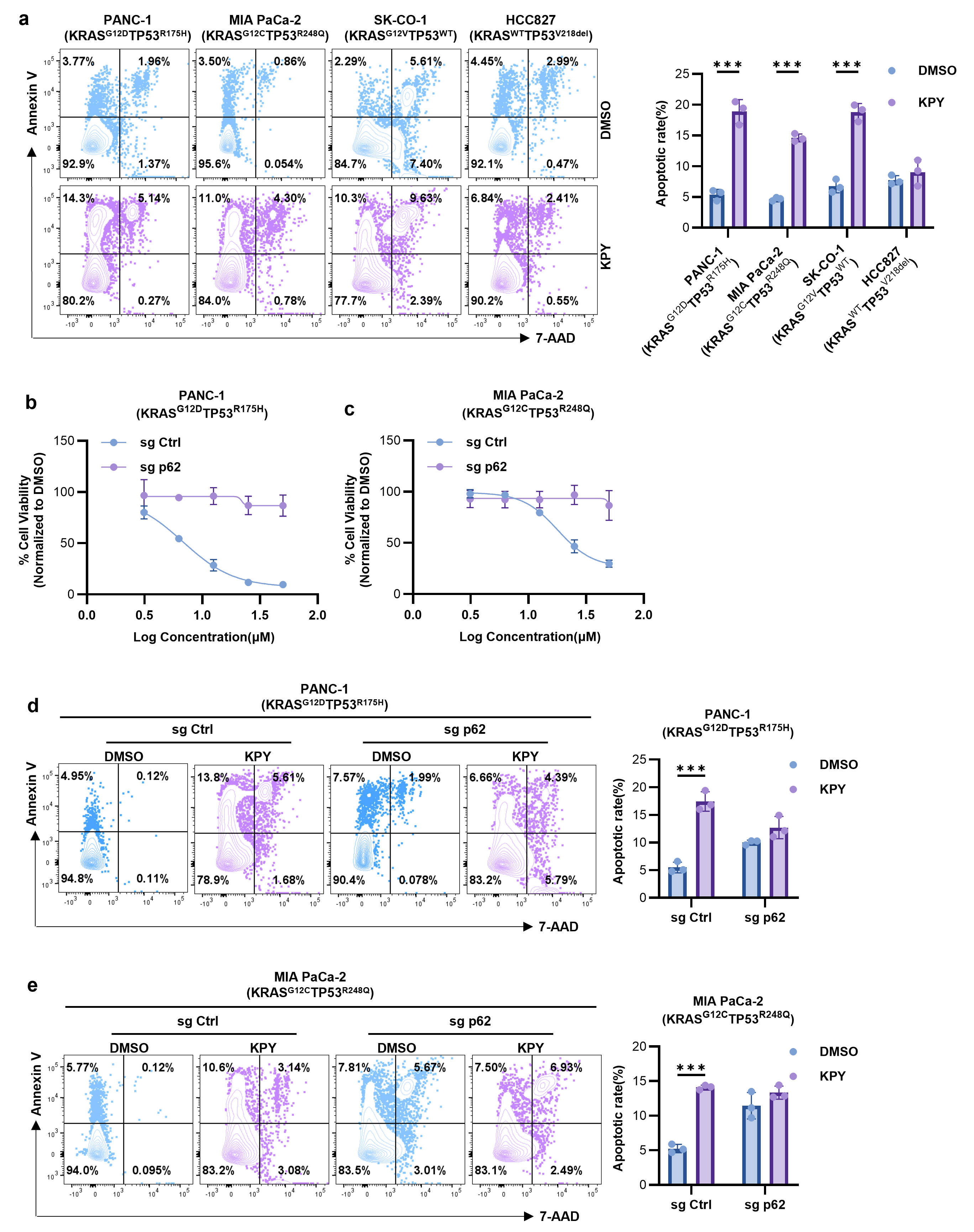
**

**Figure S6.** Cytotoxic effects of KPY on tumor cells. a) Apoptosis in different cell lines treated with DMSO or KPY (20 μM) for 48 h, measured by flow cytometry (n = 3 per group). b-c) Cell viability of PANC-1 (b) and MIA PaCa-2 cells (c) with sgRNA-mediated p62 knockdown following treatment with the indicated concentrations of KPY for 48 h, measured by the CCK-8 assay (n = 3 per group). d-e) Apoptosis in PANC-1 (d) and MIA PaCa-2 cells (e) with sgRNA-mediated p62 knockdown following treatment with DMSO or KPY (20 μM) for 48 h, measured by flow cytometry (n = 3 per group). Data are presented as mean ± s.d.; statistical significance was assessed by two-way ANOVA (a, d, e); ***P < 0.001.


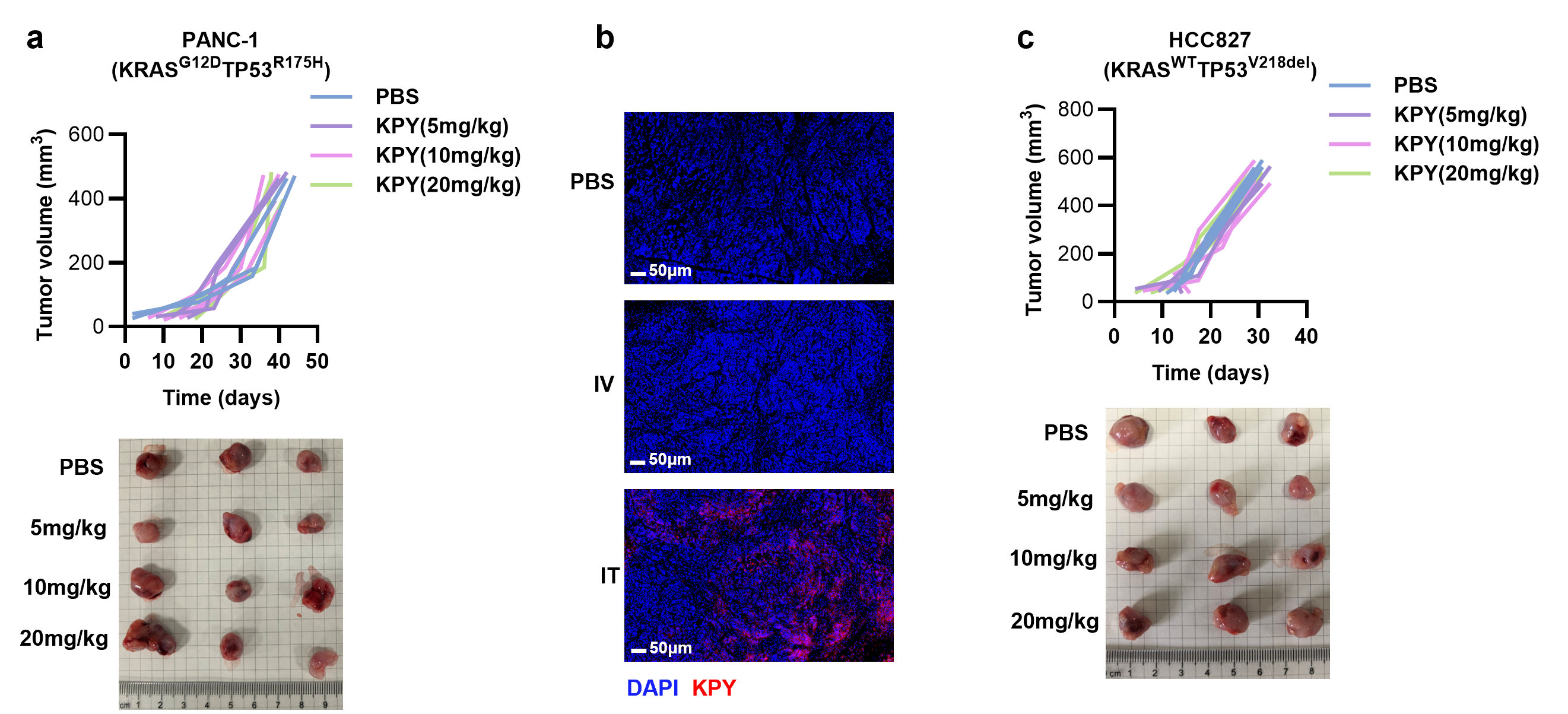


**Figure S7.** Antitumor efficacy of KPY *in vivo*. a) Tumor growth curves and tumor images for PANC-1 xenografts treated with intravenous PBS or KPY at the indicated doses (n = 3 per group). b) Representative immunofluorescence images of intratumoral KPY infiltration in PANC-1 tumors at 48 h after treatment. c) Tumor growth curves and tumor images for HCC827 xenografts treated with intratumoral PBS or KPY at the indicated doses (n = 3 per group). Data are presented as mean ± s.d.

**
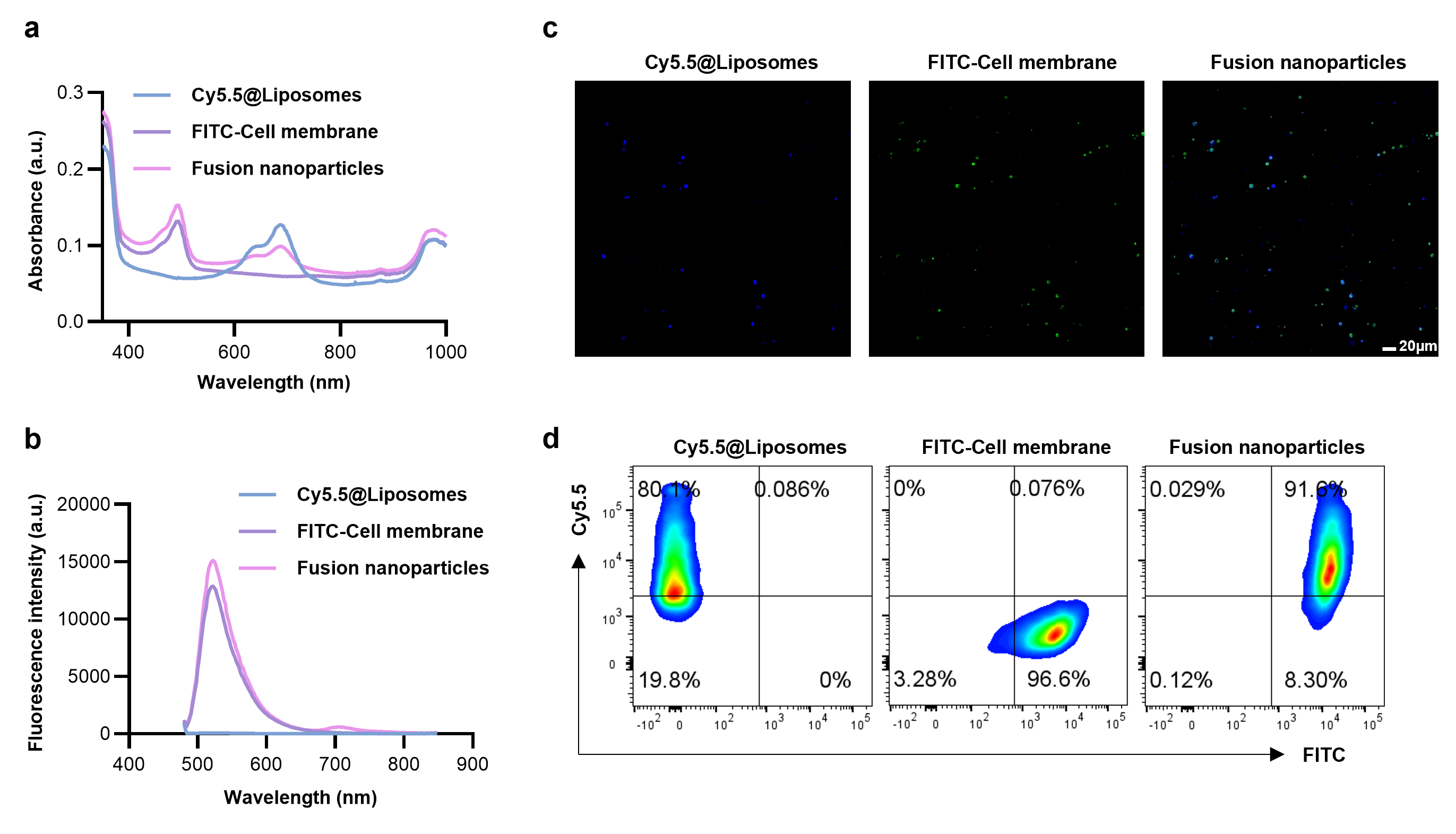
Figure S8.** Assessment of membrane–liposome fusion efficiency. a) Fluorescence spectra of Cy5.5-labeled liposomes, FITC-labeled cell membranes, and their fused nanoparticles. b) Fluorescence resonance energy transfer images of Cy5.5-labeled liposomes, FITC-labeled cell membranes, and their fused nanoparticles. c) Representative confocal microscopy images of Cy5.5-labeled liposomes, FITC-labeled cell membranes, and their fused nanoparticles. d) Flow cytometric analysis of the fusion efficiency between Cy5.5-labeled liposomes and FITC-labeled cell membranes.


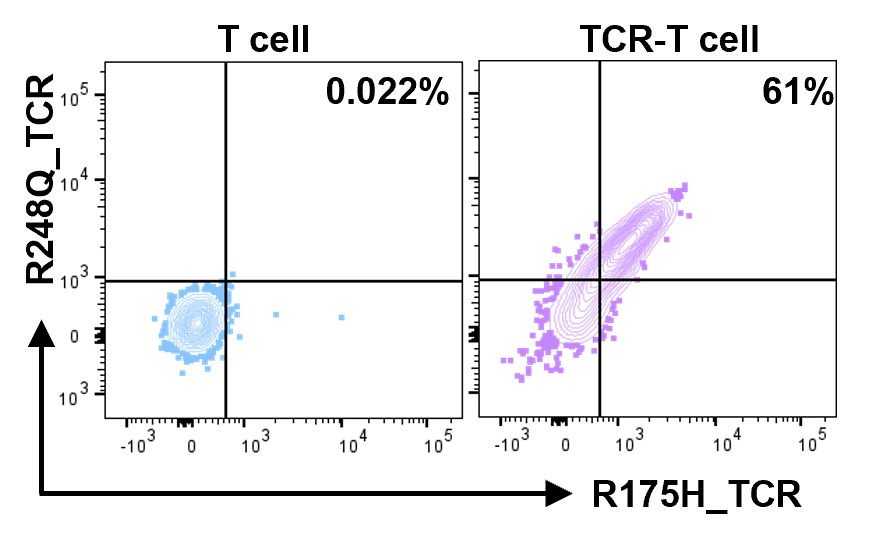


**Figure S9.** Flow-cytometric detection of TP53 R175H and R248Q TCR expression in TCR-T cells.


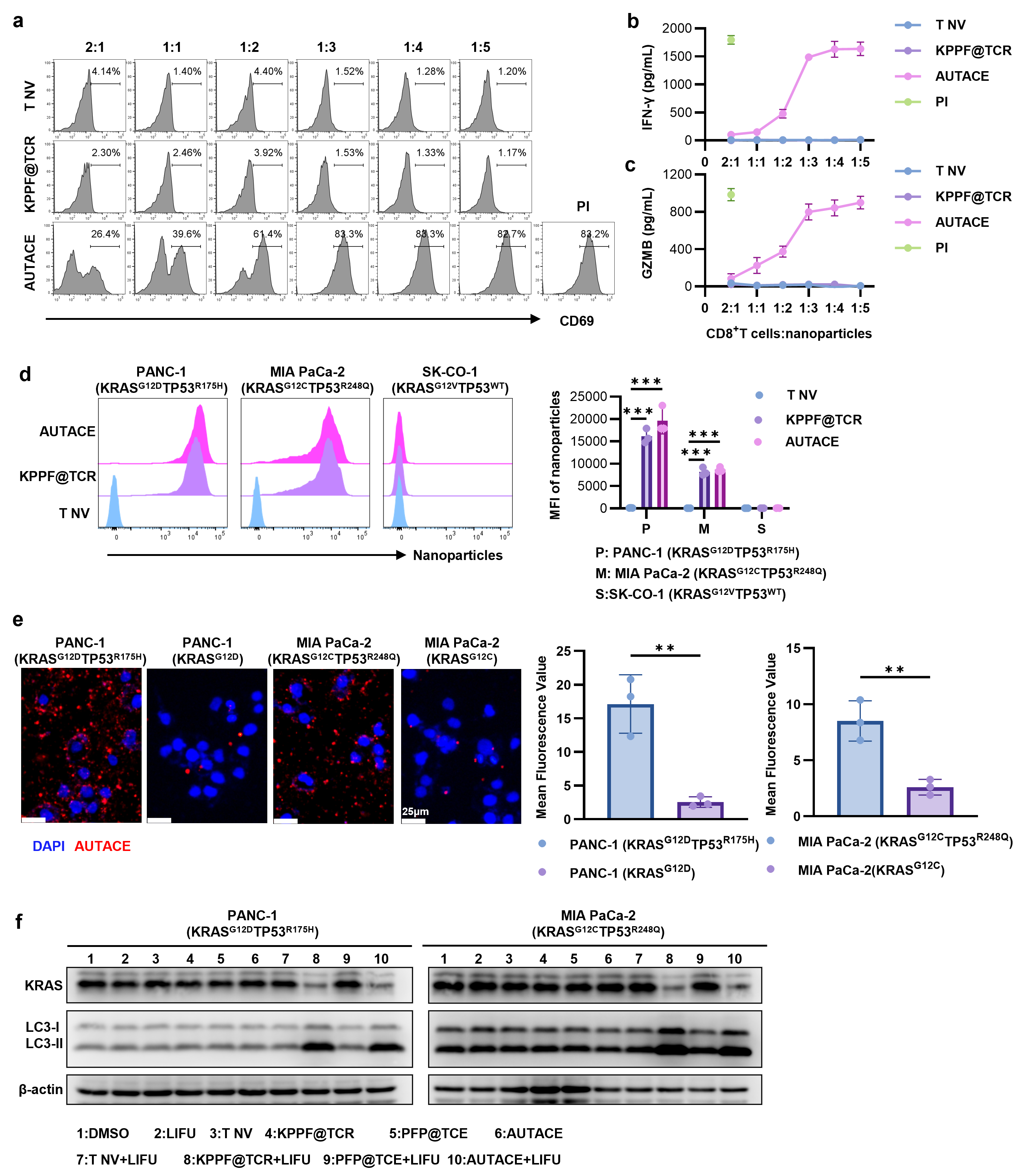


**Figure S10.** In vitro functional evaluation of AUTACE. a-c) CD8⁺ T cells were incubated with the indicated nanoparticles for 24 h, CD69 expression was quantified by flow cytometry (a), and an Enzyme-linked immunosorbent assay measured IFN-γ (b) and GzmB (c) levels in the supernatants (n = 3 per group). d) Flow-cytometric analysis of the binding of the indicated nanoparticles to PANC-1, MIA PaCa-2, and SK-CO-1 cells (n = 3 per group). e) Representative confocal microscopy images and corresponding quantitative analysis of the binding of the AUTACE to PANC-1 cells expressing or lacking the TP53 R175H mutation and to MIA PaCa-2 cells expressing or lacking the TP53 R248Q mutation (n = 3 per group). f) Western blot analysis of KRAS, LC3, and β-actin in PANC-1 and MIA PaCa-2 cells after the indicated nanoparticle-based treatments (3 mg/mL), with or without LIFU (1 W/cm² for 5 min), for 24 h; representative images from one of three independent experiments are shown.T NV: liposomes fused with plasma membranes from T cells lacking TCR expression. KPPF@TCR: KPY- and PFP-loaded liposomes fused with TCR-T cell plasma membranes. PFP@TCE: PFP-loaded liposomes fused with TCR-T cell plasma membranes and conjugated with anti-CD3 antibodies. AUTACE: KPPF@TCR further conjugated with anti-CD3 antibodies. Data are presented as mean ± s.d.; statistical significance was assessed by two-way ANOVA (d) and t-tests (e); **P < 0.01, ***P < 0.001.


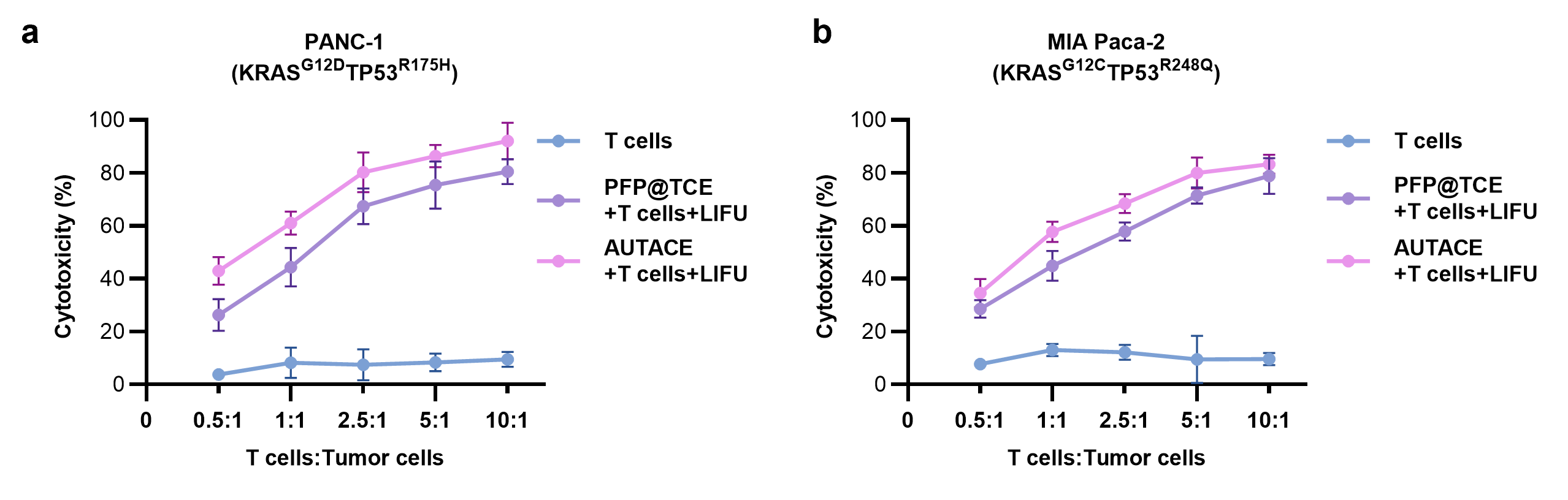


**Figure S11.** Synergistic antitumor effects of AUTACE and CD8^+^T cells. a-b) The survival of PANC-1 (a) and MIA PaCa-2 (b) cells was assessed by luciferase activity after 24 h of co-incubation with CD8⁺ T cells and the indicated nanoparticle-based treatments (3 mg/mL), with or without LIFU (1 W/cm² for 5 min) as indicated (n = 3 per group). KPPF@TCR: KPY- and PFP-loaded liposomes fused with TCR-T cell plasma membranes. PFP@TCE: PFP-loaded liposomes fused with TCR-T cell plasma membranes and conjugated with anti-CD3 antibodies. AUTACE: KPPF@TCR further conjugated with anti-CD3 antibodies. For western blot data, representative images from one of three independent experiments are shown. Data are presented as mean ± s.d.


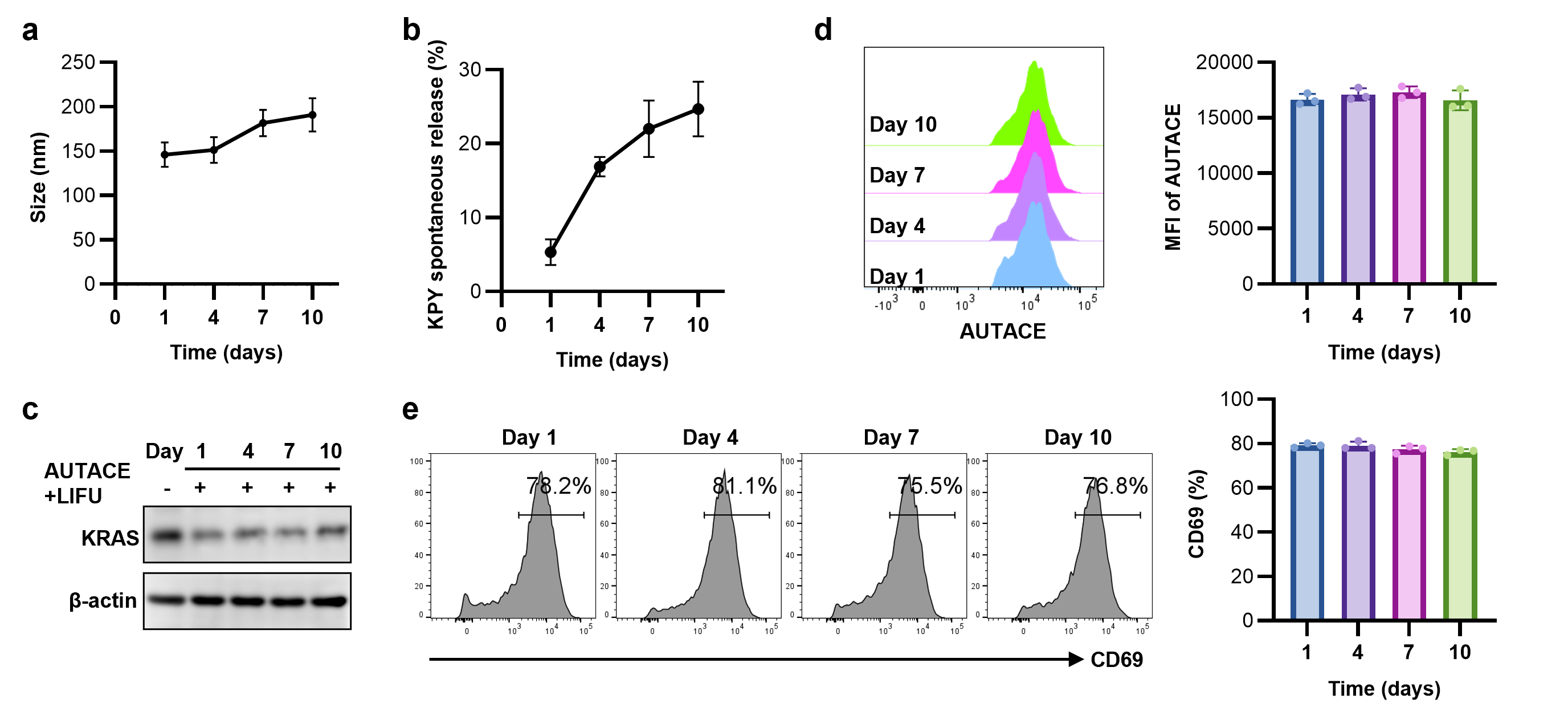


**Figure S12.** The stability of AUTACE. a) Hydrodynamic size of AUTACE after storage in PBS at 4℃ for different periods of time (n = 3 per group). b) Cumulative spontaneous release of KPY from AUTACE after storage in PBS for different periods of time (n = 3 per group). c) Western blot analysis of KRAS and β-actin in PANC-1 cells treated for 24 h with LIFU in combination with AUTACE (3 mg/mL) that had been stored in PBS for different periods of time; representative images from one of three independent experiments are shown. d) Flow cytometric analysis of the binding of AUTACE stored in PBS for different periods of time to PANC-1 cells (n = 3 per group). e) Flow cytometric analysis of CD69 expression in CD8⁺ T cells treated with AUTACE (3 mg/mL) that had been stored in PBS for different periods of time (n = 3 per group).Data are presented as mean ± s.d.; statistical significance was assessed by one-way ANOVA (d, e).


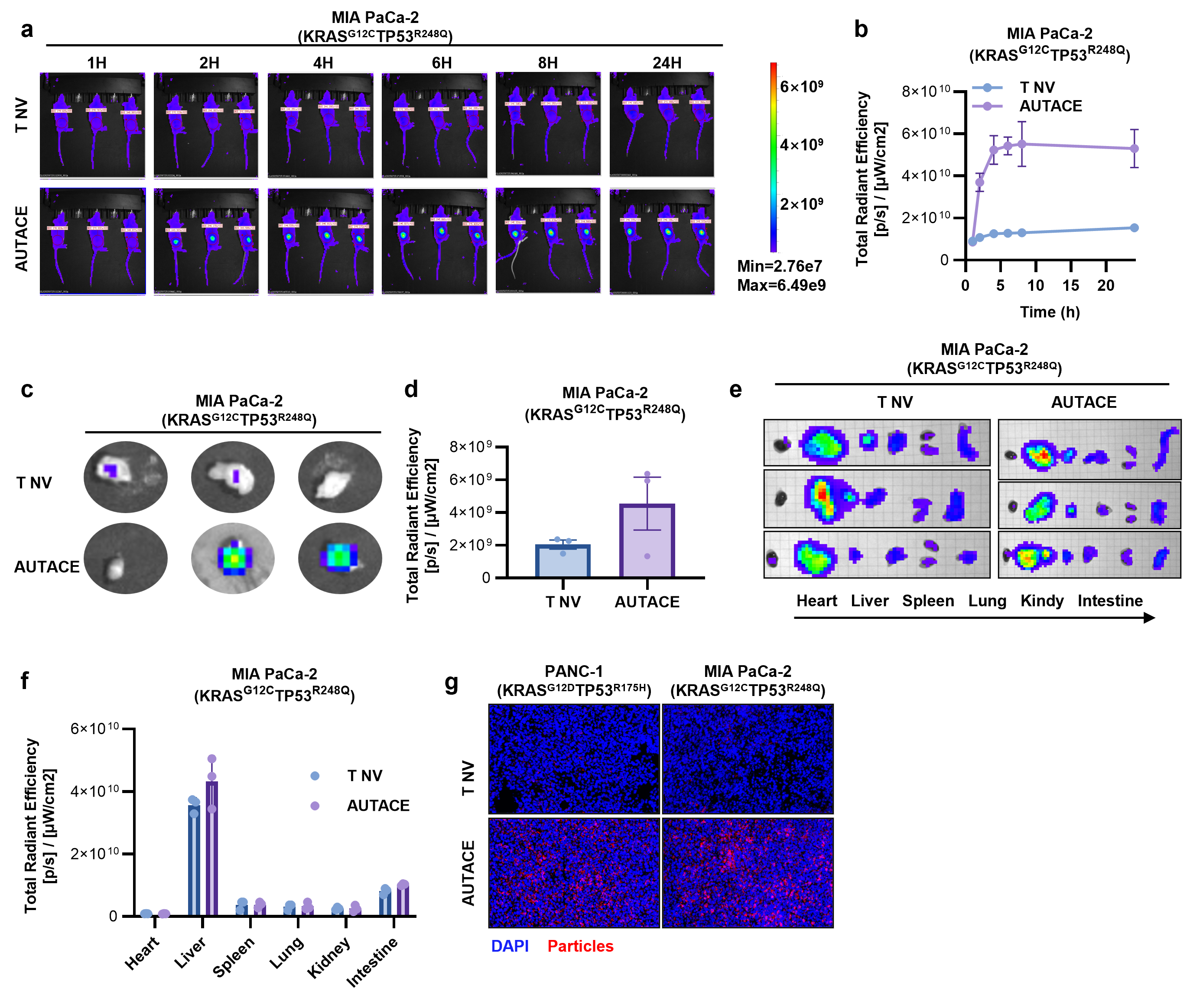


**Figure S13.** Biodistribution of AUTACE in MIA PaCa-2 tumor-bearing mice. a-b) Whole-body fluorescence imaging (a) and quantitative analysis of tumor-site fluorescence intensity (b) at the indicated time points after tail-vein injection of DiD-labeled T NV or AUTACE (50 mg/kg) in NOG mice bearing MIA PaCa-2 tumors (n = 3 per group). c-d) Ex vivo fluorescence images of resected MIA PaCa-2 tumors at 48 h after treatment (c) and corresponding quantitative analysis of fluorescence intensity (d) (n = 3 per group). e-f) Ex vivo fluorescence images of resected organs at 48 h after treatment (e) and corresponding quantitative analysis of fluorescence intensity (f) (n = 3 per group). g) Representative immunofluorescence images of intratumoral nanoparticle infiltration in PANC-1 and MIA PaCa-2 tumors at 48 h after treatment. T NV: liposomes fused with plasma membranes from T cells lacking TCR expression. KPPF@TCR: KPY- and PFP-loaded liposomes fused with TCR-T cell plasma membranes. AUTACE: KPPF@TCR further conjugated with anti-CD3 antibodies. Data are presented as mean ± s.d.; statistical significance was assessed by t-tests (d) and two-way ANOVA (f).


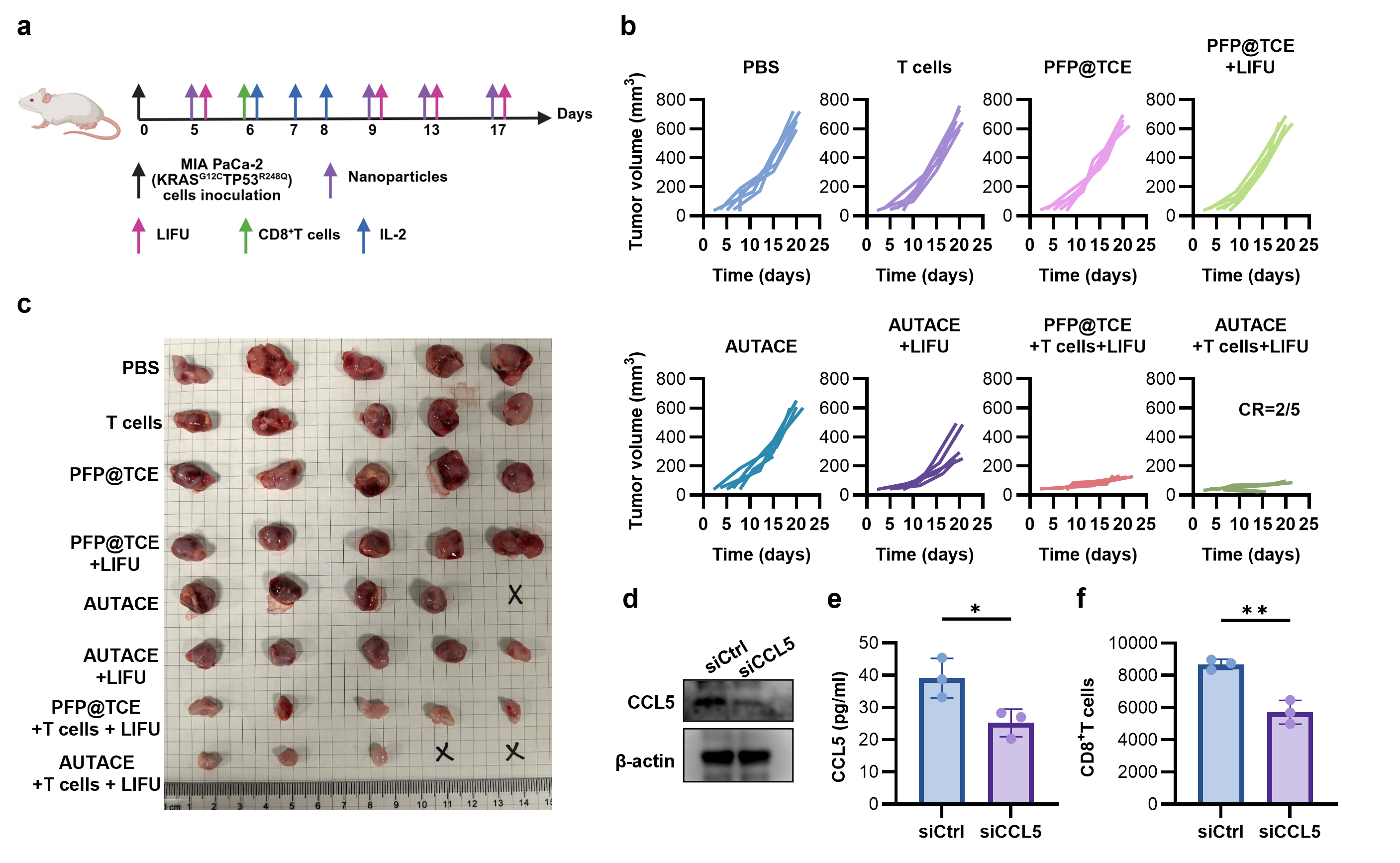


**Figure S14.** Antitumor eﬀect of AUTACE in MIA PaCa-2 tumor-bearing mice. a) Treatment schedule for the in vivo antitumor study, including nanoparticle-based treatments (50 mg/kg), LIFU irradiation (1 W/cm² for 15 min), IL-2 administration (2 × 10^5 U), and adoptive transfer of CD8⁺ T cells (1 × 10^7 cells). b-c) Individual tumor growth curves (b) and tumor images (c) of MIA PaCa-2 tumors after various treatments (n = 5 per group). d) Western blot analysis of CCL5 and β-actin in PANC-1 cells with siRNA-mediated CCL5 knockdown; representative images from one of three independent experiments are shown. e) Enzyme-linked immunosorbent assay quantification of CCL5 in culture supernatants from PANC-1 cells with siRNA-mediated CCL5 knockdown after treatment with AUTACE (3 mg/mL) plus LIFU (1 W/cm² for 5 min) (n = 3 per group). f) Flow cytometric quantification of CD8⁺ T cells that migrated across transwell inserts in response to the conditioned medium shown in (e) (n = 3 per group). KPPF@TCR: KPY- and PFP-loaded liposomes fused with TCR-T cell plasma membranes. PFP@TCE: PFP-loaded liposomes fused with TCR-T cell plasma membranes and conjugated with anti-CD3 antibodies. AUTACE: KPPF@TCR further conjugated with anti-CD3 antibodies. Data are presented as mean ± s.d.; statistical significance was assessed by t-tests (e, f); *P < 0.05, **P < 0.01.


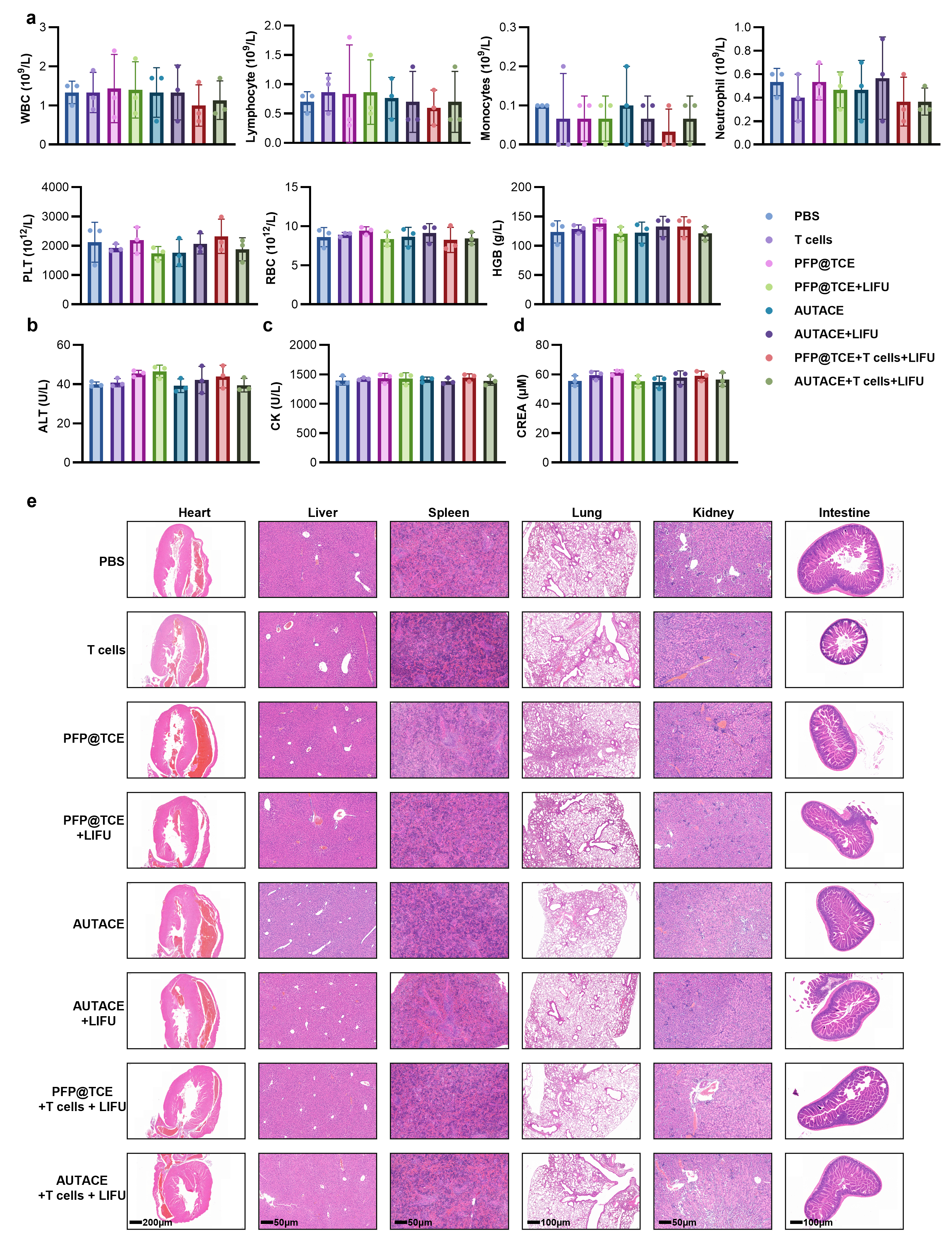


**Figure S15.** In vivo safety assessment of AUTACE. a-c) Hematologic parameters (a) and serum levels of alanine aminotransferase (b), creatine kinase (c), and creatinine (d) in PANC-1 tumor–bearing mice at day 21 after tumor inoculation in the indicated treatment groups (n = 3 per group). e) Representative H&E-stained histological images of the heart, liver, spleen, lung, kidney, and intestine from PANC-1 tumor–bearing mice at day 21 after tumor inoculation in the indicated treatment groups. KPPF@TCR: KPY- and PFP-loaded liposomes fused with TCR-T cell plasma membranes. PFP@TCE: PFP-loaded liposomes fused with TCR-T cell plasma membranes and conjugated with anti-CD3 antibodies. AUTACE: KPPF@TCR further conjugated with anti-CD3 antibodies. Data are presented as mean ± s.d.; statistical significance was assessed by one-way ANOVA (a, b, c, d).
