# Supplementary material for: The AUTACE That Degrades KRAS and Engages CD8+ T Cells for the Treatment of KRAS/TP53 Co‐Mutant Tumors
Source: Adv Sci (Weinh). 2026 Apr 7:e18455. Online ahead of print. doi: 10.1002/advs.202518455 (PMC13334621; doi:10.1002/advs.202518455)
Supplement: Supplementary file 2 — Supporting File: advs75219‐sup‐0002‐TableS1.docx. [file ADVS-9999-e18455-s002.docx]

| Parameter | T NV | KPPF@TCR | PFP@TCE | AUTACE |
| --- | --- | --- | --- | --- |
| Size (nm) | 102.29 ± 9.85 | 145.53 ± 12.66 | 115.76 ± 11.65 | 153.27 ± 28.35 |
| PDI | 0.131 ± 0.015 | 0.122 ± 0.010 | 0.256 ± 0.020 | 0.121 ± 0.014 |
| Zeta potential (mV) | -23.21 ± 1.02 | -27.19 ± 2.64 | -13.25 ± 2.62 | -16.98 ± 1.24 |
| Anti-CD3 (copies/vesicle) | None | None | 17099.67 ± 1692.14 | 16454.6 ± 2452.41 |
| CVs of anti-CD3 | None | None | 57.1 % ± 0.66% | 59.63 % ± 3.96% |
| R248Q_TCR (copies/vesicle) | None | 12563.57 ± 956.45 | 12257.25 ± 944.72 | 12778.41 ± 569.14 |
| CVs of R248Q_TCR | None | 52.23% ± 2.15% | 52.83% ± 1.59% | 51.2 % ± 2.6% |
| R175H_TCR (copies/vesicle) | None | 11195.01 ± 924.05 | 11040 ± 996.56 | 10701.62 ± 1234.66 |
| CVs of R175H_TCR | None | 54.63% ± 1.65% | 53.03 % ± 1.53% | 53.33 % ± 0.67% |

**Supplementary Table 1.** Characterization of nanoparticles
