# Supplementary material for: The AUTACE That Degrades KRAS and Engages CD8+ T Cells for the Treatment of KRAS/TP53 Co‐Mutant Tumors
Source: Adv Sci (Weinh). 2026 Apr 7:e18455. Online ahead of print. doi: 10.1002/advs.202518455 (PMC13334621; doi:10.1002/advs.202518455)
Supplement: Supplementary file 3 — Supporting File: advs75219‐sup‐0003‐TableS2.docx. [file ADVS-9999-e18455-s001.docx]

**Supplementary Table 2. Histological analysis of the Organ**

| Group | Heart | | | | Liver | | | | Spleen | | | | Lung | | | | Kidney | | | | Intestine | | | |
| --- | --- | --- | --- | --- | --- | --- | --- | --- | --- | --- | --- | --- | --- | --- | --- | --- | --- | --- | --- | --- | --- | --- | --- | --- |
|  | A | B | C | D | A | B | C | D | A | B | C | D | A | B | C | D | A | B | C | D | A | B | C | D |
| PBS | ND | ND | ND | NL | ND | ND | ND | NL | ND | ND | ND | NL | ND | ND | ND | NL | ND | ND | ND | NL | ND | ND | ND | NL |
| T cells | ND | ND | ND | NL | ND | ND | ND | NL | ND | ND | ND | NL | ND | ND | ND | NL | ND | ND | ND | NL | ND | ND | ND | NL |
| [PFP@TCE](mailto:PFP@TCE) | ND | ND | ND | NL | ND | ND | ND | NL | ND | ND | ND | NL | ND | ND | ND | NL | ND | ND | ND | NL | ND | ND | ND | NL |
| [PFP@TCE+LIFU](mailto:PFP@TCE+LIFU) | ND | ND | ND | NL | ND | ND | ND | NL | ND | ND | ND | NL | ND | ND | ND | NL | ND | ND | ND | NL | ND | ND | ND | NL |
| AUTACE | ND | ND | ND | NL | ND | ND | ND | NL | ND | ND | ND | NL | ND | ND | ND | NL | ND | ND | ND | NL | ND | ND | ND | NL |
| AUTACE+LIFU | ND | ND | ND | NL | ND | ND | ND | NL | ND | ND | ND | NL | ND | ND | ND | NL | ND | ND | ND | NL | ND | ND | ND | NL |
| [PFP@TCE+T cells +LIFU](mailto:PFP@TCE+T+LIFU) | ND | ND | ND | NL | ND | ND | ND | NL | ND | ND | ND | NL | ND | ND | ND | NL | ND | ND | ND | NL | ND | ND | ND | NL |
| AUTACE+T cells +LIFU | ND | ND | ND | NL | ND | ND | ND | NL | ND | ND | ND | NL | ND | ND | ND | NL | ND | ND | ND | NL | ND | ND | ND | NL |

A: Necrosis; B: Edema; C: Hemorrhage; D: Leukocyte Infiltration; ND: Not Detected; NL: Normal
